# Predicted Shifts in Small Mammal Distributions and Biodiversity in the Altered Future Environment of Alaska: An Open Access Data and Machine Learning Perspective

**DOI:** 10.1371/journal.pone.0132054

**Published:** 2015-07-24

**Authors:** A. P. Baltensperger, F. Huettmann

**Affiliations:** EWHALE Lab, Department of Biology and Wildlife, Institute of Arctic Biology, University of Alaska Fairbanks, Fairbanks, Alaska, United States of America; Ecologie, Systématique & Evolution, FRANCE

## Abstract

Climate change is acting to reallocate biomes, shift the distribution of species, and alter community assemblages in Alaska. Predictions regarding how these changes will affect the biodiversity and interspecific relationships of small mammals are necessary to pro-actively inform conservation planning. We used a set of online occurrence records and machine learning methods to create bioclimatic envelope models for 17 species of small mammals (rodents and shrews) across Alaska. Models formed the basis for sets of species-specific distribution maps for 2010 and were projected forward using the IPCC (Intergovernmental Panel on Climate Change) A2 scenario to predict distributions of the same species for 2100. We found that distributions of cold-climate, northern, and interior small mammal species experienced large decreases in area while shifting northward, upward in elevation, and inland across the state. In contrast, many southern and continental species expanded throughout Alaska, and also moved down-slope and toward the coast. Statewide community assemblages remained constant for 15 of the 17 species, but distributional shifts resulted in novel species assemblages in several regions. Overall biodiversity patterns were similar for both time frames, but followed general species distribution movement trends. Biodiversity losses occurred in the Yukon-Kuskokwim Delta and Seward Peninsula while the Beaufort Coastal Plain and western Brooks Range experienced modest gains in species richness as distributions shifted to form novel assemblages. Quantitative species distribution and biodiversity change projections should help land managers to develop adaptive strategies for conserving dispersal corridors, small mammal biodiversity, and ecosystem functionality into the future.

## Introduction

The world’s biomes, specifically in the Arctic and boreal forest of the circumpolar North are undergoing dramatic changes in climate, geographic distribution, ecosystem function, and food web structure [1–5]. The implications of these types of biome-level transitions for species persistence may be profound, and without accurate spatial descriptions, predictions, and monitoring, the consequences of this change on ecological systems can scarcely be foreseen. As climate change shifts biome and ecosystem boundaries, and the expanding human footprint [6] encroaches further into wildlife habitat, species must either adapt to live within these new biological limits, disperse to regions with more favorable environmental conditions, or suffer extirpation [4, 7–8]. As species respond to environmental change, we are likely to witness novel species interactions and a rearrangement of communities that may have ecosystem-wide consequences [9–10].

Species ranges are already shifting at global decadal averages of 6.1 km towards the poles and 6.1 m upward in elevation as climate conditions release southern species from environmental constraints [8, 11]. In the Arctic, where climate effects will be especially pronounced, species turnover rates are expected to be 25–38% [3]. Biome-level transitions [12] are squeezing species distributions, especially in arctic and alpine habitats where they are often confined to increasingly smaller areas [13–15]. As the tundra warms and dries, and permafrost melts, sedge-dominated vegetation is giving way to shrubs and trees that are expanding both their latitudinal and elevational extents [14, 16–18]. Preliminary assessments show that tundra refugia are disappearing in southwestern Alaska and shrinking towards the Arctic coast north of the Brooks Range [12]. At the same time, alpine habitats are being pushed upward by rising treelines, their total area limited by the decreasing amount of land towards mountain summits [19–22]. Arctic- and alpine-adapted wildlife species are trapped in a waning biome with increasingly limited options for dispersal and persistence [4, 23–24]. These changes may adversely affect ecological functionality as the uncertain effects of species turnover cascade up the food chain [8, 21, 25–26].

In the context of these sweeping environmental changes, it is critical for the scientific conservation of species and ecosystem services to determine how the distribution and functionality of biotic systems will respond, especially in the North where changes are rapid and extensive [16, 27–28]. However, the consequences of climate-induced changes on Alaskan terrestrial food web systems, especially the bottom-up effects of prey composition, remains one of the least studied and understood fields of global change biology [13, 27, 29]. To have an efficient system of global ecological management, it is essential to understand the details of these processes.

Small mammals compose a diverse and populous set of primary and secondary consumers, and are themselves essential prey for a variety of carnivorans and raptors [15, 30–31]. Rodents (Rodentia) and shrews (Soricidae) occupy a range of niche spaces and maintain various combinations of herbivorous, frugivorous, granivorous, fungivorous, and insectivorous diets [32–34]. In Alaska, rodents provide numerous ecological services including seed dispersal [35], mycorrhizal fungal symbiosis [36], soil development [34], and herbivory [30–31, 37], whereas insectivorous shrews are known to be valuable for controlling invertebrate populations [38]. Several core communities of sympatric small mammal species exist across the state [39] with co-occurring species largely maintaining diets partitioned among different combinations of herbaceous plants and fungi [32]. However, it remains unknown whether these communities will withstand the disruptive pressures of anthropogenic climate change.

Despite general observed elevational and latitudinal trends in species movement, the exact nature of individual small mammal responses to climate change remains complex and sometimes even counterintuitive as species react to new combinations of interacting environmental and ecological conditions [8, 24, 26, 40]. Often, these processes are discovered to be more complicated than initially predicted, as additional analyses reveal new drivers and interactions that change existing assumptions. Here we use a machine-learning-based bioclimatic envelope modeling approach [41–45] to outline current environmental conditions conducive for the occurrence of small mammals in Alaska. Based on the IPCC (International Panel on Climate Change; [2]) A2 climate scenario, we project our models onto future bioclimatic conditions to outline regions likely to undergo major changes in biodiversity and community composition. These results will help to further pro-active ecological management based on the best-available science.

## Methods

### Data Collation

**Fig 1:**
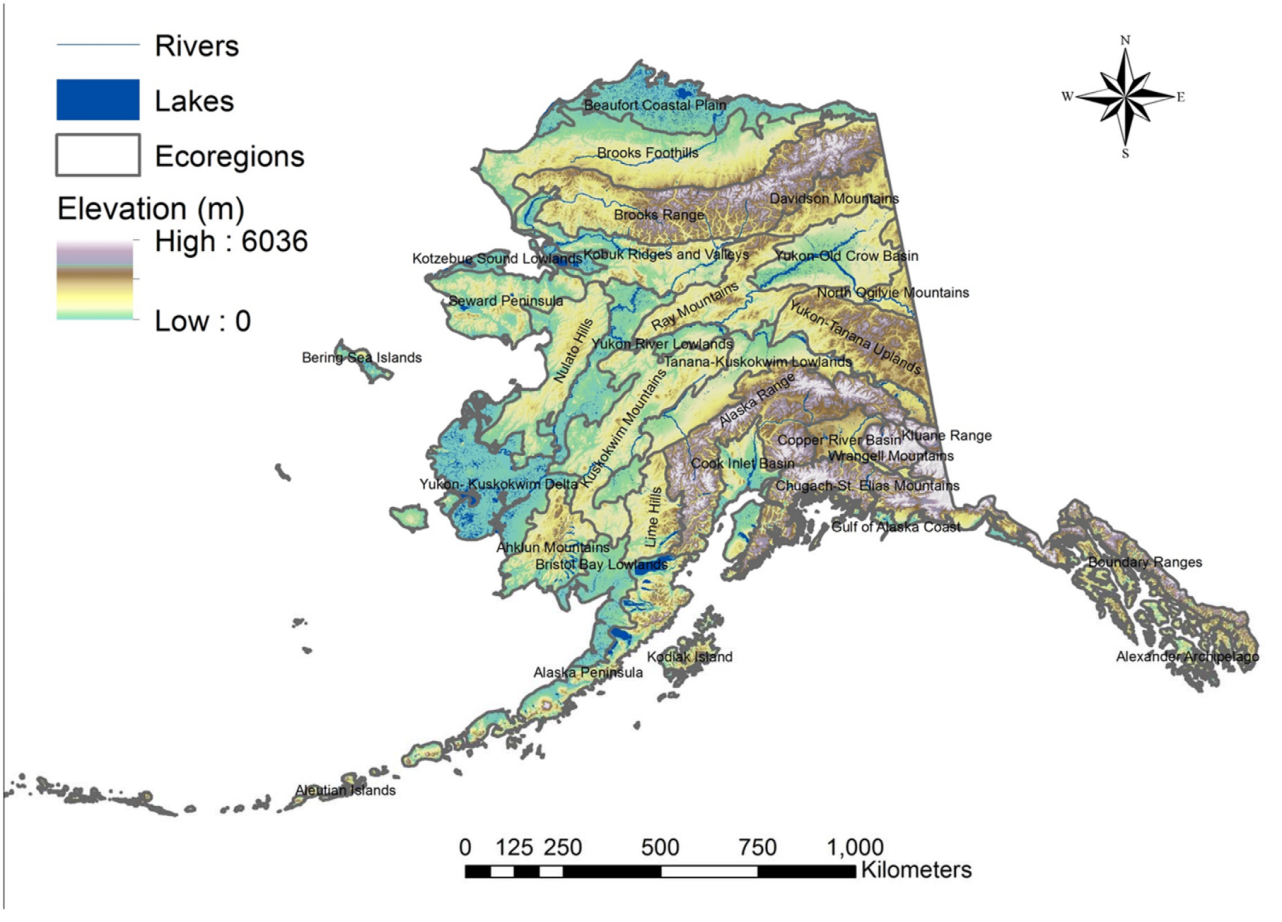
Study area map. Depiction of the study area composed of the state of Alaska. Ecoregion boundaries are shown for reference.

We compiled over 112,000 digital georeferenced records of small mammals collected from a diversity of arctic and boreal ecoregions across Alaska ([39], Fig 1, S1 Dataset). A smaller, filtered, subset of this dataset was used as training data to create distribution models for 17 species of rodents and shrews across Alaska (Table 1). Data were collated from archived occurrence datasets, primarily from the Global Biodiversity Information Facility (GBIF; www.gbif.org), and from several natural history museum collections that do not serve their data to GBIF. The compiled set of presence-only records was filtered to remove spatial duplicates and records without geographic precision to at least five decimals. Because of the presence-only nature of archived datasets that lack a geographically stratified design, we aimed to minimize the effects of sampling bias by using only one record per species within a 1-km radius of any given location. After manually removing imprecise and duplicate occurrence records, a total of 4,408 unique georeferenced small mammal records collected between 1900 and 2012 (85% were between 2000 and 2012) remained and comprised the current (2010) model training dataset.

**Table 1:** Species list and model results. List of modeled small mammal species scientific and common names, their associated Taxonomic Serial Number (TSN), the number of presence and absence locations used to train models, the resultant area under the receiver operator characteristic (AUC ROC; 0–1), the % of correctly identified presences (specificity), the % of correctly identified absences (sensitivity) and overall % error across all presences and absences.

Species	Common Name	TSN	Presences (*n*)	Absences (*n*)	AUC ROC	Specificity (%)	Sensitivity (%)	Accuracy (%)
*Clethrionomys/Myodes rutilus*	northern red-backed vole	180293	949	1157	0.94	96.6	71.9	87.7
*Dicrostonyx groenlandicus*	northern collared lemming	180328	35	2539	0.94	82.9	92.9	92.8
*Lemmus trimucronatus*	brown lemming	180320	142	2098	0.95	84.5	90.4	90.0
*Microtus longicaudus*	long-tailed vole	180299	191	2292	0.99	97.4	96.3	96.4
*Microtus miurus*	singing vole	180309	183	2153	0.98	90.7	93.7	93.5
*Microtus oeconomus*	root/tundra vole	180298	612	1029	0.94	87.4	84.3	85.4
*Microtus pennsylvanicus*	meadow vole	180297	244	1725	0.96	89.8	88.9	89.0
*Microtus xanthognathus*	yellow-cheeked/taiga vole	180301	88	2377	0.98	93.2	93.8	93.8
*Sorex cinereus*	cinereus/masked shrew	179929	818	267	0.89	93.4	59.6	85.1
*Sorex hoyi*	pygmy shrew	179946	97	1370	0.95	84.5	89.6	89.2
*Sorex monticolus*	montane/dusky shrew	179950	566	507	0.91	84.7	82.6	83.7
*Sorex palustris*	American water shrew	179933	13	1701	0.83	76.9	90.9	90.8
*Sorex tundrensis*	tundra shrew	179957	195	1071	0.95	88.2	85.6	86.0
*Sorex ugyunak*	barren-ground shrew	552509	37	1634	0.99	97.3	97.1	97.1
*Sorex yukonicus/minutissimus*	Alaska tiny shrew/Eurasian least shrew	555663	34	1610	0.95	91.2	94.0	93.9
*Synaptomys borealis*	northern bog-lemming	180323	142	1986	0.91	73.9	86.3	85.5
*Zapus hudsonius*	meadow jumping mouse	180386	72	2348	0.94	80.6	90.5	90.2

To create models with a binomial response (presence/absence) it was also necessary to generate a set of ‘pseudo-absences’ to represent areas where target species were unlikely to occur. We established that the presence of a non-target species, without the known coincidental occurrence of the target species within a 1-km radius, represented a pseudo-absence for the target species [46]. Despite potential differences in collection efforts between studies, this was the pseudo-absence option that resulted in the most accurate models and it performs as well as or better than other pseudo-absence scenarios [46–48].

### Model Development

We used RandomForests (Salford Systems, Inc., San Diego, CA, USA; www.salford-systems.com) to create spatial distribution models for each of the 17 species of mainland small mammals in Alaska. RandomForests uses a data-driven approach to describe patterns in datasets and are adept at predicting species distributions using large sets of environmental variables [49]. The resultant models can be applied across space and time [50–52], while accounting for the complex, confounding, and non-linear relationships among variables that drive ecological processes [50, 53–56]. The bioclimatic envelope modeling approach employed here assumes a stable niche that is conserved over time, allowing for robust quantitative predictions into the future [45, 57–58]. This approach does not however, account for the effects that dispersal, interspecific interactions, and intrinsic or evolutionary adaptation could have in altering the predicted consequences of a changing climate on distributions [21, 59–61].

Presence points and pseudo-absence points for each species were attributed with 27 environmental predictor layers (Table 2) using the *Extract values to multi-point* tool in ArcGIS 10.2 (ESRI, Inc., Redlands, CA, USA). Environmental predictor variables included continuous raster (60-m accuracy) and categorical polygon layers, for which spatial GIS layers were available for both the time periods of 2010–2020 and 2090–2100. Sets of ‘static’ predictor layers (those anticipated to undergo minimal change between 2010 and 2100) and ‘dynamic’ predictor layers (those undergoing substantial change as a result of climate effects) were selected based on their explanatory influence as documented in prior 2010 distribution models of small mammals ([39]; S1 File). The hypothesized and documented effects of these variables may occur directly at the ecosystem or landscape scales (e.g. habitat, proximity to resources, topography, etc), or indirectly at landscape or regional scales (e.g. climate, cliome, etc.; Table 2).

**Table 2:** Model variable list. Predictor variables used in models, type of data (categorical or continuous), and whether variables were changing or constant across time (static or dynamic). Online sources for layer downloads are also included. Continuous layers have a 60-m resolution.

Variable Name	Data Type	Temporal Stability	Source
Aspect	Continuous	Static	http://ned.usgs.gov/
Distance to Coastline	Continuous	Static	http://dnr.alaska.gov/SpatialUtility/SUC?cmd=vmd&layerid=56
Distance to Lakes	Continuous	Static	http://nhd.usgs.gov/
Distance to March Sea Ice	Continuous	Dynamic	Rogers et al. 2014
Distance to September Sea Ice	Continuous	Dynamic	Rogers et al. 2014
Distance to River	Continuous	Static	http://nhd.usgs.gov/
Distance to Village	Continuous	Static	http://www.adfg.alaska.gov/index.cfm?adfg=maps.data
Distance to Wetlands	Continuous	Static	http://www.fws.gov/wetlands/data/
Cliome	Categorical	Dynamic	https://www.snap.uaf.edu/projects/biome-shift
Elevation	Continuous	Static	http://ned.usgs.gov/
Mean Active Layer Thickness	Continuous	Dynamic	ftp://frosty.gi.alaska.edu/Out/Sergei/ALASKA_Model/GIPL1/
Mean Annual Precipitation	Continuous	Dynamic	https://www.snap.uaf.edu/tools/data-downloads
Mean Annual Soil Temperature	Continuous	Dynamic	ftp://frosty.gi.alaska.edu/Out/Sergei/ALASKA_Model/GIPL1/
Mean Annual Temperature	Continuous	Dynamic	https://www.snap.uaf.edu/tools/data-downloads
Mean January Precipitation	Continuous	Dynamic	https://www.snap.uaf.edu/tools/data-downloads
Mean January Temperature	Continuous	Dynamic	https://www.snap.uaf.edu/tools/data-downloads
Mean First Day of Freeze	Continuous	Dynamic	https://www.snap.uaf.edu/tools/data-downloads
Mean First Day of Thaw	Continuous	Dynamic	https://www.snap.uaf.edu/tools/data-downloads
Mean July Precipitation	Continuous	Dynamic	https://www.snap.uaf.edu/tools/data-downloads
Mean July Temperature	Continuous	Dynamic	https://www.snap.uaf.edu/tools/data-downloads
Mean Number of Grow Days	Continuous	Dynamic	https://www.snap.uaf.edu/tools/data-downloads
Mean January Snow Day Fraction	Continuous	Dynamic	https://www.snap.uaf.edu/tools/data-downloads
Mean July Snow Day Fraction	Continuous	Dynamic	https://www.snap.uaf.edu/tools/data-downloads
Slope	Continuous	Static	http://ned.usgs.gov/
Soil Type	Categorical	Static	http://www.nrcs.usda.gov/wps/portal/nrcs/site/ak/home/
Surficial Geology	Categorical	Static	http://agdc.usgs.gov/data/usgs/geology/metadata/beikman.html
Terrain	Continuous	Static	http://ned.usgs.gov/

The combined 2010 presence/pseudo-absence training datasets for each species were then modeled in RandomForests. Each model was grown to at least 500 trees and used all other software default settings to obtain the best-possible models. Aspatial performance was cross-validated internally in RandomForest using an ‘out-of-bag’ set of training points [53]. The discrimination capacity of each model was calculated using the area under the curve (AUC) [62] based on the receiver-operating characteristic (ROC) and predictive performance was assessed using the Overall % Correct. Both of these metrics are quantifications of correctly-predicted presences and absences in each model [54, 63–64]. A symmetric threshold of 0.5 was used to differentiate between presences and absences for all models.

To create species distribution maps for 2010 and 2100, files containing the predictive algorithm (‘groves’) were applied to a regular lattice of points (5-km resolution) spanning Alaska. Points in the lattice were also previously attributed with the environmental variable sets for 2010 and 2100. Static variables were held constant across the 2010 and 2100 models, while dynamic variables were updated for the 2100 model using decadal mean projections for 2090 to 2100 based on a five-model average of IPCC projections using the A2 emissions scenario [2]. The A2 scenario describes a heterogeneous world with a continuously increasing global population projected to reach 15 billion people by 2100 and rapid growth in carbon emissions related to land use in excess of 29 billion GtC/yr [65]. This is one of the most pessimistic emissions scenarios in the public discussion for which downscaled climate projections were available, and so models based on it represent a widely-accepted, ‘worst-case’ scenario for climate change [65]. Considering that current CO_2_ emissions have exceeded those of the A2 scenario for the past decade with little reduction likely in the near future, this scenario was a realistic choice [66].

Our model outputs generated relative indices of occurrence (RIO) for each point in the regular lattice for 2010 and 2100. RIO is a ranking of pixels from 0 to 1 representing the likelihood of belonging to the ‘presence’ class) and is not the same as a probability of occurrence at a given pixel. In ArcGIS 10.2, RIO values were smoothed for visualization between neighboring points across the study area extent using the Inverse Distance Weighting (IDW) tool with a 1-km resolution and clipped to the state coastline. This resulted in independent, spatially-continuous, predictive distribution maps for each species of small mammal in Alaska for the time periods of 2010 and 2100. RandomForests was also used to rank the relative importance of environmental variables in models.

### Community Composition Analysis

To compare community composition between the two time periods (2010 and 2100) we created a set of 50,000 random points across Alaska and attributed each point with the RIO values from the 17 future species distribution models. This provided a means for determining co-occurring species at each point. We used the chart.correlation command from the Hmisc package (F. Harrell; https://github.com/harrelfe/Hmisc) in R 2.12.1 to provide a quantified assessment of the similarities among RIO values of all species at each point. Species-pairings with correlation coefficients ≥ 0.25 were considered to be positively correlated and likely to co-occur in space, whereas pairings with a coefficient < −0.25 were negatively correlated and unlikely to co-occur. Clusters of correlated species were visualized in tree form using the varclus command in Hmisc, so that we could easily identify the main groups of sympatric species that occur together. These groups were named as the most frequent small mammal communities in Alaska. Comparisons between the tree structures of the two time periods identified how species membership among these communities are likely to change between 2010 and 2100.

### Spatio-Temporal Change Analysis

To quantify biodiversity and biogeographic patterns across Alaska, we reclassified all species distribution models to binary rasters based on a threshold of 0.5. Pixels with RIO ≥ 0.5 were classified as 1 (denoting species presence), and pixels with RIO < 0.5 were classified as 0 (denoting species absence). Binary rasters were summed across all species for each time period, yielding species richness maps for 2010 (Bio_2010_) and 2100 (Bio_2100_). Using the Raster Calculator in ArcGIS 10.2, we subtracted the species richness map for 2010 from that of 2100 to yield a map (ΔBio) depicting the projected net change in biodiversity across Alaska over the coming century. Similar calculations were used to compare continuous species RIO maps between the two time periods.

The resultant set of maps (ΔRIO_species_) depicted the projected change in relative occurrence for each species between 2010 and 2100. The mean RIO across all species was then calculated for each pixel resulting in a map (ΔRIO) that detailed the average net change in species relative occurrence for Alaska. For all species in each time period, we also calculated the median latitude, elevation and distance to the coast for pixels classified as presences. This allowed for the quantification of species distribution movement rates northward, coastward and upward in elevation.

To further analyze shifts in binary species distribution maps and to calculate the total difference in area and percent change for species distributions between 2010 and 2100, we used the Land Change Modeler (Clark Labs, Worchester, MA, USA) in ArcGIS 10.2. Resultant maps depicted areas of loss, gain and persistence for each species. The regular grid of 5-km spaced points across Alaska was also attributed with the pixel values of the four composite species models (Bio_2010_, Bio_2100_, ΔBio, and ΔRIO). The attributed lattice was then analyzed in TreeNet (Salford Systems, Inc., San Diego, CA, USA; www.salford-systems.com) to determine the importance rankings of variables in each model.

## Results

### Future Distribution and Community Models

Models predicting the distributions of 17 species of small mammals (S1 Fig) were highly accurate, evaluated using withheld out-of-bag samples (Table 1). All models exceeded 85% overall accuracy except for the montane shrew (*Sorex monticolus*; 83.7%). When evaluated using the AUC ROC scores, all species exceeded 0.89 with the exception of the American water shrew (*S*. *palustris*; 0.83).

**Fig 2:**
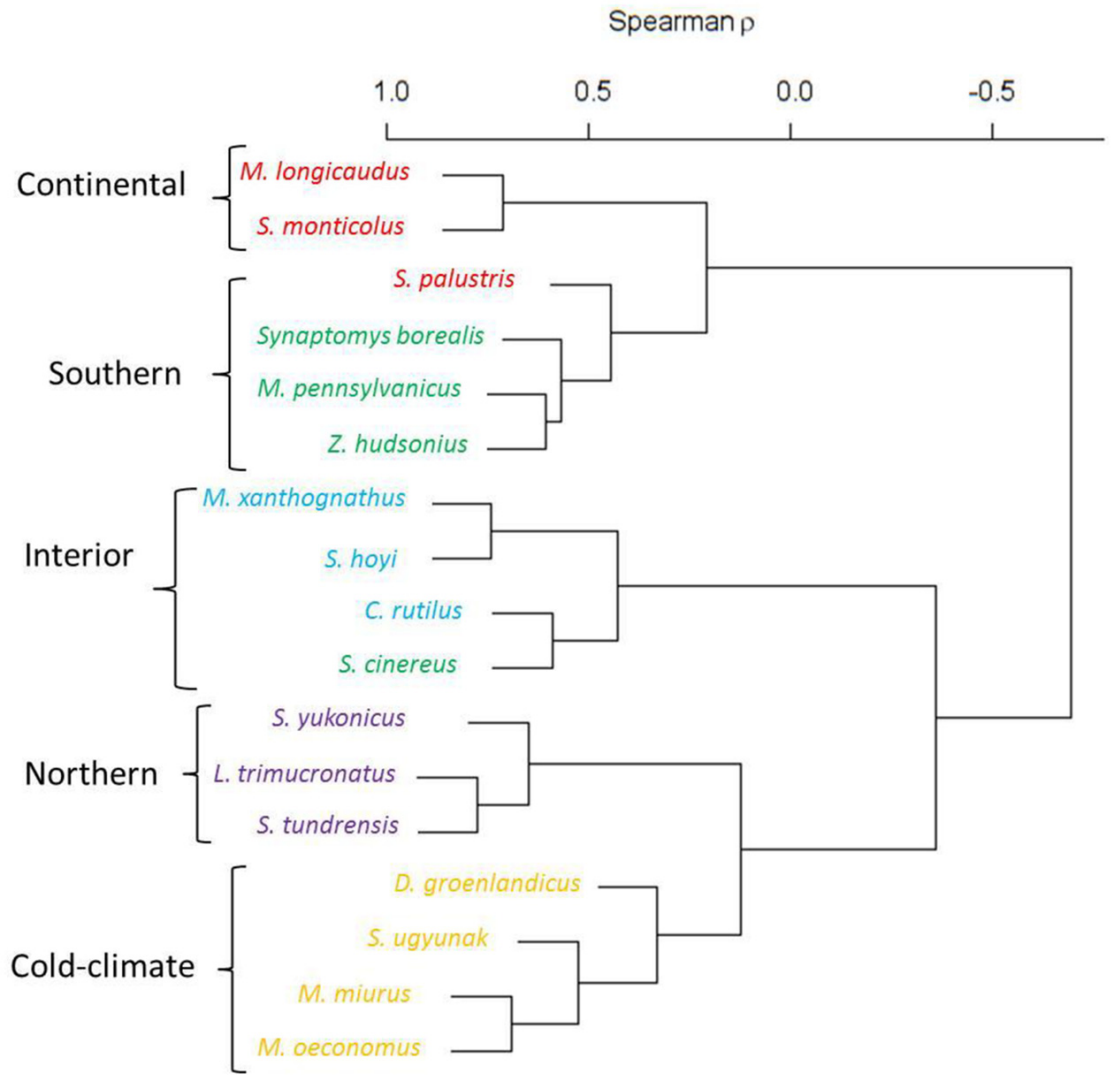
*Varclus* correlation tree. Projected 2100 community arrangements for 17 species of small mammals in Alaska based on a varclus correlation analysis in R. Brackets aggregate species into 2100 community groups, while colors indicate species membership in 2010 community groups.

Projected future distributions of small mammals were grouped into five distinct community groups across Alaska using varclus. Community groups were remarkably similar in structure to those calculated for 2010 and included the same ‘continental’, ‘southern’, ‘interior’, ‘northern’, and ‘cold-climate’ aggregations ([39], S1 File). Species membership within groups remained the same between 2010 and 2100 with the exception that cinereus shrews (*Sorex cinereus*) moved from the southern community to the interior community and American water shrews moved from the continental community to the southern community (Fig 2), reflecting a northward and interior contraction of the cinereus shrew distribution and a broad expansion of the water shrew distribution. The continental and southern communities were positively correlated with one another, but negatively correlated with all other groups. Likewise, the northern and cold-climate groups were also positively correlated, but were negatively correlated with all other groups.

### Model Change between 2010 and 2100

Comparisons between current and future species distribution models showed an average loss in area of 20.2% for all species in the cold-climate, northern, and interior community groups (Table 3). Among these groups, only the pygmy shrew (*S*. *hoyi*) experienced increases in total area (3.5%). In contrast, distributions of all species in the continental and southern communities increased by an average of 29.2%, with the only exception being that the area occupied by meadow voles (*Microtus pennsylvanicus*) decreased by 3.5%.

**Table 3:** Species model change metrics. Total predicted areas of presence for each of 17 species of Alaskan small mammals in 2010 and 2100. Net change is the 2010 area subtracted from that of 2100. % change is the number of pixels changed in the presence class divided by the area of the presence class for 2100. Changes in latitude, distance to coast, and elevation were calculated by subtracting the median value in 2100 from that of 2010. Negative values for latitude, coast distance, and elevation indicate southerly, coastward, and downslope shifts, respectively.

Species	Presence Area 2010	Presence Area 2100	Net Δ (km^2^)	% Δ	Latitude Δ (km)	Coast Distance Δ (km)	Elevation Δ (m)	Community
*Dicrostonyx groenlandicus*	603,960	550,725	-53,235	-6.0	-25	8.3	66.8	cold-climate
*Microtus miurus*	589,108	377,223	-211,885	-4.3	130	-3.0	167.0	cold-climate
*Microtus oeconomus*	1,083,164	823,741	-259,423	-4.8	105	44.9	88.5	cold-climate
*Sorex ugyunak*	412,527	198,763	-213,764	-19.7	85	-6.0	-65.8	cold-climate
**Mean**	**672,190**	**487,613**	**-184,577**	**-8.7**	**73.7**	**11.0**	**64.1**	**cold-climate**
*Microtus longicaudus*	206,803	336,130	129,327	10.0	35.0	6.7	32.9	continental
*Sorex monticolus*	335,761	382,230	46,469	4.0	-595.0	-147.9	108.2	continental
**Mean**	**271,282**	**359,180**	**87,898**	**7.0**	**-280.0**	**-70.6**	**70.6**	**continental**
*Clethrionomys/Myodes rutilus*	803,289	609,189	-194,100	-31.9	135.0	9.3	105.2	interior
*Microtus xanthognathus*	355,644	219,628	-136,016	-11.9	45.0	31.7	75.9	interior
*Sorex cinereus*	1,192,694	1,105,717	-86,977	-28.5	50.0	11.7	3.7	interior
*Sorex hoyi*	607,161	637,943	30,782	3.5	130.0	5.9	20.7	interior
**Mean**	**739,697**	**643,119**	**-96,578**	**-17.2**	**90.0**	**14.7**	**51.4**	**interior**
*Lemmus trimucronatus*	702,596	395,636	-306,960	-38.6	210.0	19.7	40.5	northern
*Sorex tundrensis*	867,006	455,138	-411,868	-65.3	280.0	2.0	5.8	northern
*Sorex yukonicus/minutissimus*	418,908	259,669	-159,239	-14.8	75.0	-11.6	-47.2	northern
**Mean**	**662,837**	**370,148**	**-292,689**	**-39.6**	**188.3**	**3.4**	**-0.3**	**northern**
*Microtus pennsylvanicus*	335,399	294,218	-41,181	-3.5	-90.0	-71.3	-0.9	southern
*Sorex palustris*	237,571	1,049,427	811856	64.4	85.0	-0.9	-202.4	southern
*Synaptomys borealis*	532,151	979,025	446,874	46.3	-50.0	-62.9	-19.8	southern
*Zapus hudsonius*	438,181	1,010,635	572,454	54.0	155.0	12.1	115.2	southern
**Mean**	**385,826**	**833,326**	**447,501**	**40.3**	**25.0**	**-30.8**	**-27.0**	**southern**

Distributions of all members of the cold-climate, northern, and interior communities also showed northward shifts in median latitude that averaged 111 km using the implemented modeling scenario. Only northern collared lemmings (*Dicrostonyx groenlandicus*) experienced a southerly shift of 25 km (2.8 km/decade) by 2100. Latitudinal changes for the continental and southern communities were mixed, with half of the species moving north and the other half moving south. Average upward shifts of 46.3 m in elevation (5.1 m/decade) were observed for species in the cold-climate, northern, interior and continental communities, whereas members of the southern community decreased by an average of 74.4 m (8.3 m/decade) in elevation. Only barren-ground shrews (*S*. *ugyunak*) and Alaska tiny shrews (*S*. *yukonicus*; but see [67] for taxonomy) moved downslope whereas meadow jumping mice (*Zapus hudsonius*) moved upslope, contradicting the elevational trends of their respective community groups.

Changes in median distance to the coast were more varied. The distributions of all interior community species moved further inland by an average of 14.7 km (1.6 km/decade), as did brown lemmings (*Lemmus trimucronatus*) and tundra shrews (*S*. *tundrensis*) from the northern community. Species distributions in the southern community (except meadow jumping mice) shifted closer to the coast, but responses among cold-climate and continental community species were equally divided in their movement relative to the coast (Table 3).

**Fig 3:**
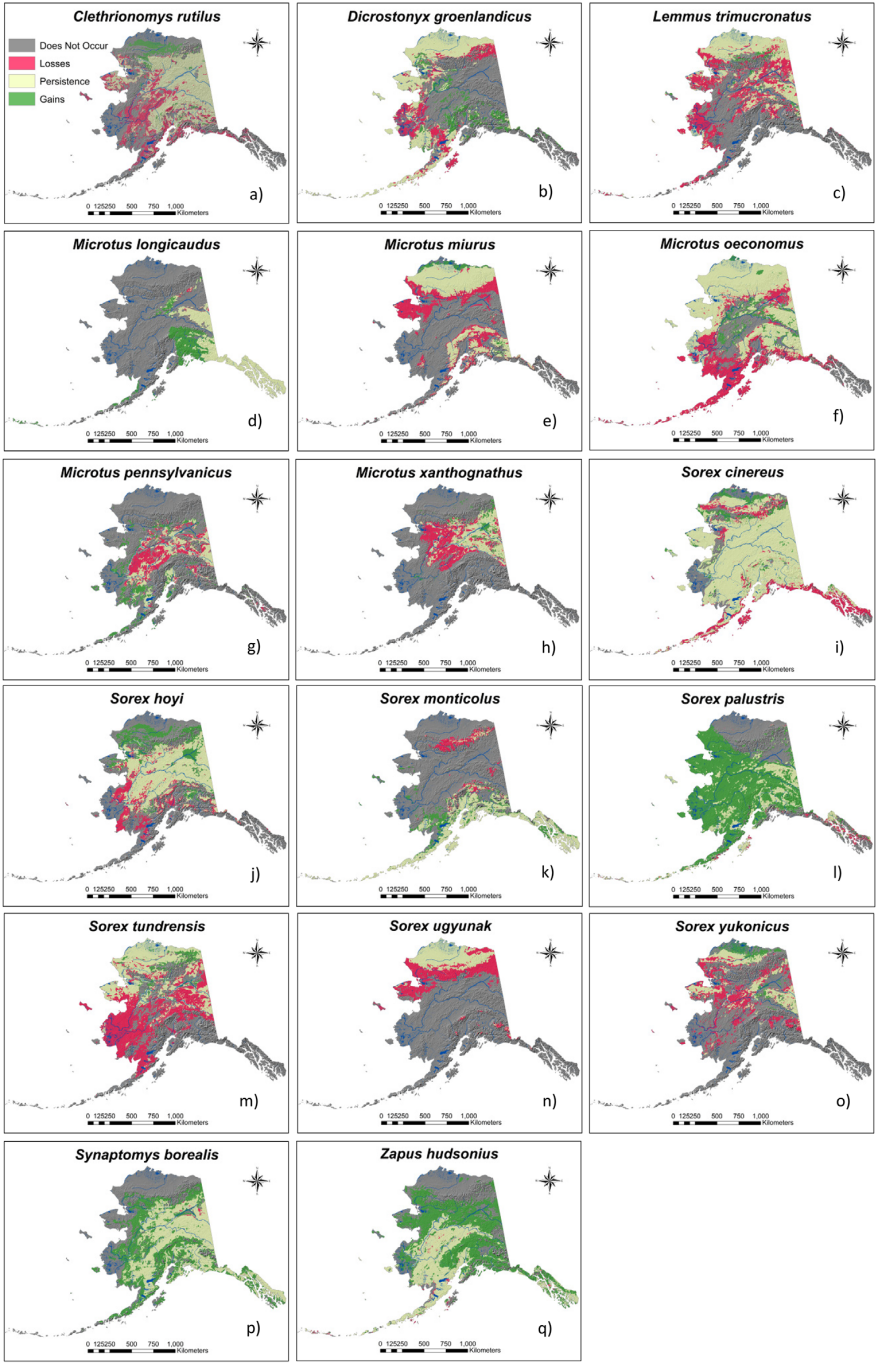
Distribution change maps. Predicted distribution change for each of the 17 modeled species of small mammal in Alaska: a) northern red-backed vole (*Clethrionomys rutilus*), b) northern collared lemming (*Dicrostonyx groenlandicus*), c) brown lemming (*Lemmus trimucronatus*), d) long-tailed vole (*Microtus longicaudus*), e) singing vole (*M*. *miurus*), f) root vole (*M*. *oeconomus*), g) meadow vole (*M*. *pennsylvanicus*), h) yellow-cheeked vole (*M*. *pennsylvanicus*), i) cinereus shrew (*Sorex cinereus*), j) pygmy shrew (*S*. *hoyi*), k) montane shrew (*S*. *monticolus*), l) American water shrew (*S*. *palustris*), m) tundra shrew (*S*. *tundrensis*), n) barren-ground shrew (*S*. *ugyunak*), o) Alaska tiny shrew (*S*. *yukonicus*), p) northern bog-lemming (*Synaptomys borealis*), q) meadow jumping mouse (*Zapus hudsonius*). Red = areas of distribution loss, green = areas of distribution gain, and yellow = areas of persistence.

Land Change Modeler analyses highlighted core regions of persistence as well as areas of distribution loss and gain that were similar among each community’s members (Fig 3). Among species of the cold-climate and northern communities, models showed major areas of distribution loss to occur in the Davidson Mountains, on the Seward Peninsula, and in the Yukon-Kuskokwim (Y-K) Delta. Similarly, interior community species distributions were reduced or eliminated from the southern extents of their ranges especially on the Alaska Peninsula and in southeast Alaska. Northern red-backed vole (*Clethrionomys rutilus*; but see [68] for taxonomy) and yellow-cheeked vole (*M*. *xanthognathus*) distributions were also projected to shift inland across the western portions of their ranges. Most species in these communities were also predicted to experience distribution contractions at low to mid elevations in the Brooks Range and the mountain ranges of southcentral Alaska (Fig 3). Minor distribution gains were predicted at the northern distribution extents of some species on the Beaufort Coastal Plain.

Distributions of species in the southern community were projected to gain an average of 40.3% in area between 2010 and 2100. Specifically, meadow jumping mice and American water shrews more than doubled the size of their distributions (Table 3). Species in this community expanded westward into the Y-K Delta, Nulato Hills, and Seward Peninsula and northward into the Alaska and Brooks Ranges. Continental community species distributions were also predicted to extend westward into mountainous regions of interior and south-central Alaska, and in the case of montane shrews (*S*. *monticolus*) as far as the Alaska Peninsula (Fig 3). Projected distribution losses for these communities were minimal but included the disappearance of water shrews from southeast Alaska, meadow voles from the Kuskokwim Mountains and montane shrews from the Brooks Range.

### Changes in Small Mammal Biodiversity

**Fig 4:**
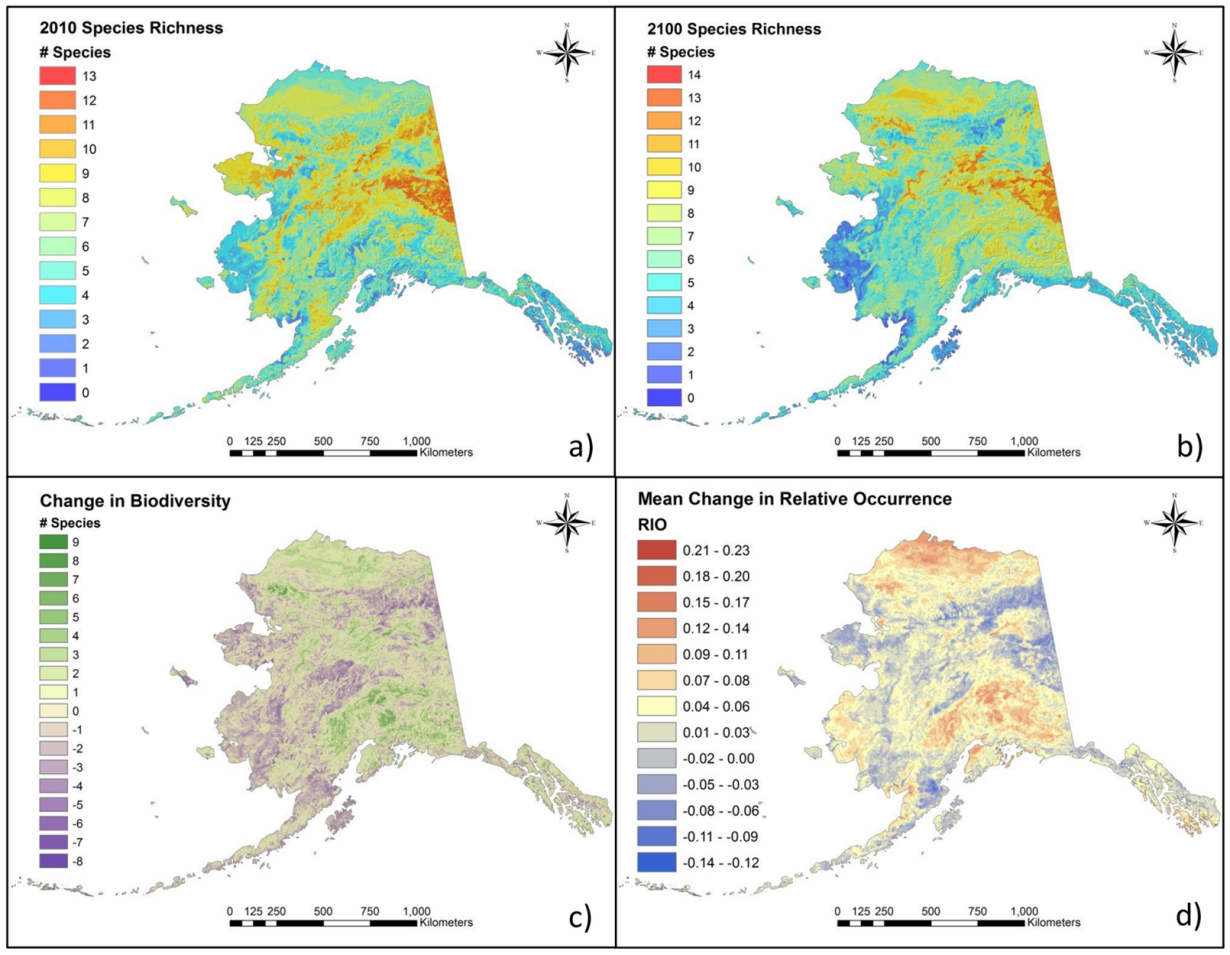
Species richness change maps. Predictive species richness maps based on composites of binary (presence/absence) maps for 17 species of small mammals for the years a) 2010 (modified from [39]; S1 File) and b) 2100. Maps also depict net change in c) species richness (ΔBio) and d) relative indices of occurrence (ΔRIO). Warm colors indicate net gains in RIO (relative index of occurrence) and species richness, whereas cool colors indicate net loss of RIO and species richness.

**Fig 5:**
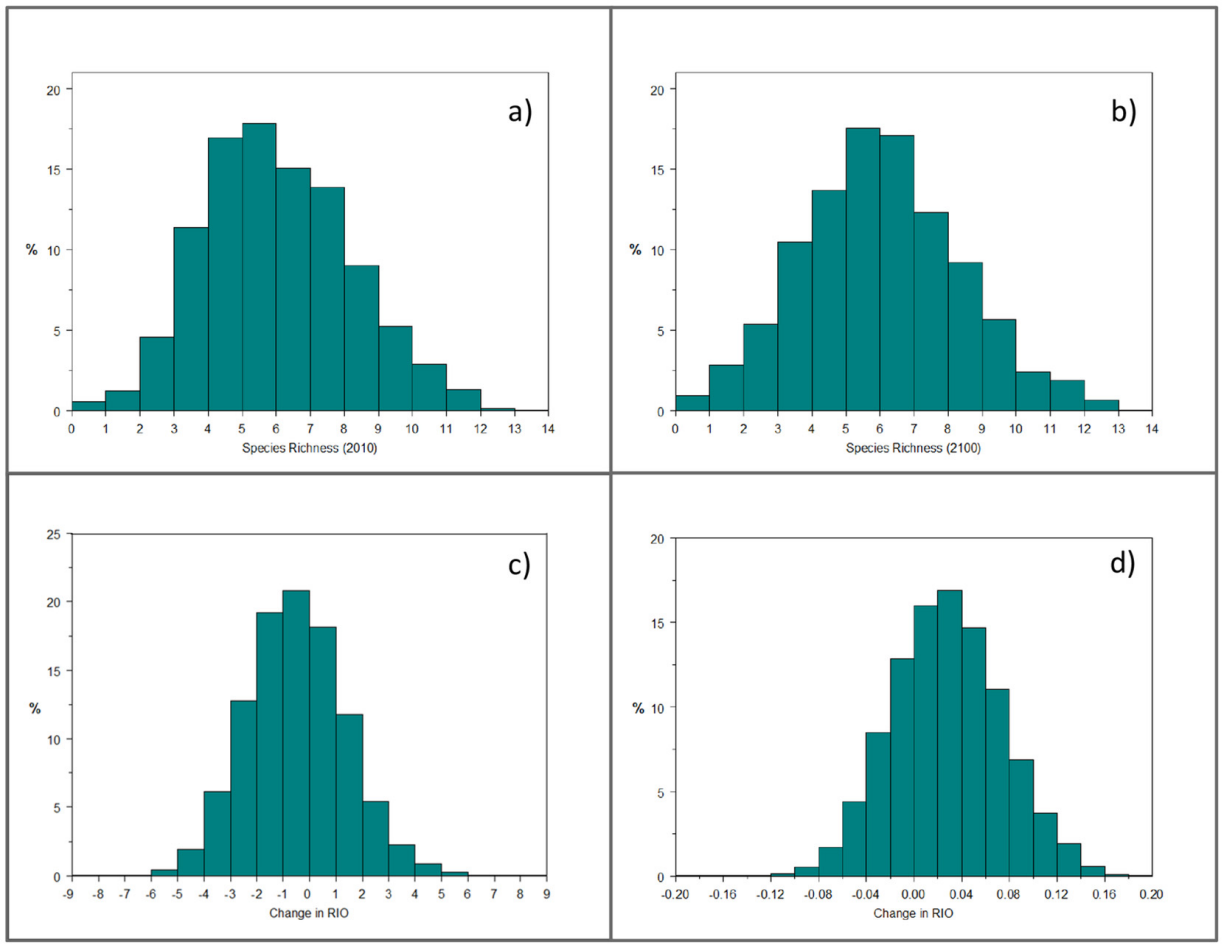
Species richness graphs. Histograms depicting the frequency of pixels for the number of species in a) 2010, b) 2100, as well as the net change in c) species richness, and d) relative indices of occurrence.

Geographic patterns of predicted small mammal biodiversity in 2100 were similar to those in 2010 (Fig 4). Biodiversity ‘hot-spots’ with the highest species richness (14 species) in 2100 were predicted for 10 km^2^ of the Brooks Range and Ogilvie Mountains and 13 species were predicted to occur in 765 km^2^ of mountainous boreal forest scattered throughout the upland regions of central Interior Alaska (Fig 4). The lowest small mammal species richness (≤ 3 species) was predicted for southwest Alaska and the eastern Brooks Range (Fig 4). Changes in species richness that were ± 1 or less occurred in 59% of the state, however some regions experienced changes in overall small mammal diversity as high as +9 and as low as −8 species (Figs 4 and 5). A small decrease in overall biodiversity occurred between 2010 and 2100, yet areas containing 4 to 5 species in 2010 underwent an increase of 1 to 2 species (Fig 5). Areas of increased biodiversity were projected for mountainous regions as well as at lower elevations north of the Brooks Range. Top explanatory variables accounting for these patterns included *Soil Type*, *Surficial Geology*, *Distance to Sea Ice*, *Elevation*, *Distance to Coast*, and *Mean Active Layer Thickness*.

Analysis using mean RIO values highlighted potential movement into and out of regions, showing an overall increase in relative occurrence of species (Fig 5). The largest increases (0.23) occurred on the Beaufort Coastal Plain, in south-central Alaska, and in the Y-K Delta (Fig 4). The largest decreases in mean RIO (−0.14) occurred in the Davidson Mountains, and on the Seward and Alaska Peninsulas. Additionally, the Yukon-Tanana Uplands, saw broad decreases in mean RIO despite high species richness there. Top explanatory variables in this composite model were similar to the other composite models with the addition of *Mean Number of Growing Days*. All GIS models and predictor layers were archived and are available on the online data repository ScholarWorks@UA at the University of Alaska Fairbanks Elmer E. Rasmuson Library.

## Discussion

Extensive projected environmental changes across the state resulted in considerable directional shifts in the distributions of small mammals (Fig 3). Distributions of most members of the northern, cold-climate, and interior small mammal communities were projected to decrease in area, shift northward, upward in elevation, and away from the coast, whereas the opposite was true for many members of the southern and continental species (Table 3). While extreme rearrangements of communities were not predicted, novel patterns of species sympatry in some regions were apparent (Fig 2). Principal species assemblages remained robust at a statewide scale and shifted mainly together as units, following the movement of bioclimatic envelopes and biomes. However, novel species interactions were predicted to occur at middle elevations in mountainous areas where a combination of varied topography and overlapping distributions provided for high species richness. Biodiversity losses occurred across southwestern Alaska and in central interior Alaska, as well as on the Seward Peninsula and in the eastern Brooks Range (Fig 4). Areas with the largest species gains and the highest potential for novel species interactions occurred in the mountain ranges of southcentral Alaska, in the western Brooks Range, and across the Beaufort Coastal Plain (Fig 4).

### Distribution Shifts

Shifts in the distributions of species as well as changes in species richness are occurring northward, upward in elevation, and away from the coast. The latitudinal and elevational trends are consistent with theory and other studies that have shown the global northward and upslope movement of species as they follow their bioclimatic envelopes [11, 23, 69–70]. Despite average northward shifts in the distributions of most species, high elevations at southern latitudes of Alaska are projected to preserve pockets of tundra as potential refugia for some cold-climate species such as singing voles (*M*. *miurus*) or collared lemmings [12]. As the lowland tundra biome transitions, cold-climate and northern species currently residing there could either move upslope to alpine tundra refugia or remain at low elevations and follow the tundra biome northward. However, both alternatives depend on uncertain species-specific capacities for upslope and poleward dispersal that exceed the pace of climate change [24, 61].

Indications that most species distributions will move inland (especially in southwestern Alaska) are somewhat counterintuitive to the general trends of northward and upward movement of bioclimatic envelopes. This shift in small mammal distributions corresponds to predictions that the western tundra biome will be converted into the warmer and drier ‘boreal transition’ and ‘montane cordillera’ biomes as portions of this region may experience up to three biome state changes over the coming century [12]. Although interior community species might seem to benefit from warmer, drier conditions, they are nevertheless projected to undergo contractions in the western and coastal portions of their ranges. Not only is the western tundra biome predicted to disappear from southwestern Alaska by 2100, so too is the boreal forest biome. Just as boreal species are encroaching on arctic species at mid-elevations and higher latitudes, southern and continental community species are predicted to replace interior boreal species in southwest Alaska.

Projected distribution changes modeled here are similar to but less extreme than those predicted by other small mammal studies conducted for the Arctic [4, 23, 71–72]. Distribution models for 2010 corresponded closely with current distribution estimates for most species [23, 71]. Hope et al.’s [23] results outline analogous northward and upslope shifts in ranges that are more extreme (especially in the central Beaufort Coastal Plain) than predicted by our models. Nevertheless, patterns showing the loss of many species from the Y-K Delta and Seward Peninsula as well as increasingly overlapping distributions between boreal and arctic species are consistent between model sets [23]. Circumpolar distribution change models for collared lemmings predicted the loss of half of the current area of viable habitat by 2080 [4], while models for the Canadian Arctic projected losses of 60% given a 4°C rise in temperature by 2070 [72]. We predicted losses of just 6% in total collared lemming distribution by 2100, which sharply contradicts other results, but may be explained by modest habitat gains predicted for southern mountain ranges that were not predicted by Prost et al. [4].

### Biodiversity Change

Patterns in species richness hot- and cold-spots in 2100 indicated the Yukon-Tanana Uplands to be an important biodiversity crossroads while the Y-K Delta was predicted to have some of the lowest species richness values in the state. These patterns were similar to those predicted for 2010 [39]. But biodiversity hot-spots in 2100 were also predicted to shift northward, inland, and upward in elevation achieving the largest gains in southern mountain ranges and on the Beaufort Coastal Plain. These are the areas most likely to experience new species interactions. As temperatures warm, areas at lower elevations will become suitable for new species (mostly from the expanding southern community) at a rate that exceeds the loss of cold-adapted species already living there [23].

Evidence from other studies has indicated that the highest small mammal species diversity often occurs at mid-elevations [73–75]. Here, the addition of new species to low elevation habitats will likely push native species upslope resulting in overall increases in species richness in areas with diverse topography. Given the increasingly limited land area towards most mountain peaks, the concentration of species at mid to high elevations may be termed ‘alpine squeeze’. However, because some mountain ranges exhibit increasing topographic complexity with increasing elevation (at least toward some threshold), the area of habitat available at mid elevations may actually be larger than at more extreme elevations [76]. These effects may ultimately lead to new species contacts between invading and resident species [10, 77].

The scenario is similar on the Beaufort Coastal Plain, but there an ‘arctic squeeze’ will confine an increasing number of species to a shrinking area [15, 72]. Models predict the addition of northern red-backed voles, cinereus shrews, singing voles, and Alaska tiny shrews to the Beaufort Coastal Plain, but only indicate range contraction (not extirpation) of native species there (e.g., collared lemmings, brown lemmings, tundra shrews, and barren ground shrews). This condensed arrangement is likely to result in at least a transitional confluence of species before resident arctic species are extirpated [15, 72].

The largest losses in species richness were predicted for the western tundra ecosystems of the Y-K Delta and Seward Peninsula. Importantly, these areas currently contain some of the last lowland tundra south of the Brooks Range [4, 7, 12, 23]. The disappearance of this habitat type from those regions would spell the eventual extirpation of cold-climate and northern species such as collared lemmings, brown lemmings, singing voles, root voles, tundra shrews, and barren-ground shrews from the southern portions of their ranges.

### Important Model Predictors

Static, categorical variables that interacted strongly with many other predictors, explained a large amount of variation in the models, while dynamic climate-related variables were also important for accounting for change over time. Average variable importance rankings indicated the categorical variable, *Soil Type*, to be the single most useful variable for determining small mammal distribution and biodiversity patterns. This importance may be somewhat misleading however, as machine learning algorithms tend to inflate the importance of predictors with many categories. Nevertheless, the fact that the models are largely driven by static variables, may have served to dampen the influence of climate on species distribution and biodiversity changes, resulting in more conservative distribution change predictions.

Secondarily important variables included many dynamic climate variables such as *Distance to September Sea Ice* and *Distance to March Sea Ice*, as well as *Mean Active Layer Thickness*, and *Growing Days*. Because the top variables were static across model years, differences between the 2010 and 2100 models were mainly the result of changes in dynamic variables and their interactions with static variables. For example, the consistently high variable importance rankings of both *Distance to Sea Ice* variables underscore the effect that large-scale losses in sea ice in combination with temperature increases will have in driving distributions and community compositions for a variety of terrestrial and marine species.

### Community Structure

Community structure composition in 2100 remained akin to results from 2010, despite broad geographic and elevational shifts in the distributions of all modeled species. While theory predicts a rearrangement of community assemblages with changing environmental conditions [4, 7], our results showed that community groups responded similarly to changing climate envelopes at the statewide scale. These similarities may stem from our models not accounting for varied, species-specific adaptive capacity that would result in more diverse responses, but they also support the notion of niche conservatism. Some research suggests that niche dimensions are largely conserved at time scales shorter than those during which evolution operates [58, 78–79]. More recent research indicates that individual responses to climate change will vary based on the phenotypic and behavioral plasticity, dispersal ability, and adaptive capacity of each species over time [21, 60, 80–81].

Only two species (American water shrew and cinereus shrew) of 17 altered their community membership and both exemplify broader trends in species turnover in Alaska. A very large predicted increase in the distribution of water shrews during the coming century was out of proportion with changes experienced by other species. Because of inherent difficulties in detection, American water shrews are currently documented from only a handful of locations in Alaska. It is important to note however, that because of the small sample for water shrews, the occupied geographic niche space is likely under-sampled. This may have resulted in the low AUC ROC score and specificity for this model. As such, the extreme range expansion observed over 21^st^ century might be tempered using a larger, more representative training sample and we encourage specific efforts to detect new records of this species in Alaska. Because predictive models based on small, constrained training sets may result in artificially lowered AUC values [82], values for our models may be underestimates and as such, one must consider multiple metrics (e.g., specificity and sensitivity) when evaluating model performance.

The expansion of southern and continental species supports the notion that species from the south will continue to disperse into Alaska [72]. Because this analysis only considered resident species currently in Alaska, it is also likely that the Alaskan fauna of 2100 will include an entirely new set of non-resident species from continental North America. Future field monitoring over the coming decades in southeast Alaska and the Yukon-Tanana Uplands can help document any such invasions.

Cinereus shrews, which previously belonged to the southern community, were classified in 2100 as members of the interior community and symbolize another front in Alaskan species invasion. Unlike water shrews, but similar to other members of the interior community, the distribution of cinereus shrews is predicted to decrease by 29% and constrict toward the interior of the state. But despite losses in the southern portion of their range, they are also predicted to expand at the northern extent. The invasion of interior/boreal species into the Arctic will likely produce another zone of novel interaction and species turnover [23].

Comparisons of individual models also showed expansions of southern and continental species into areas where novel species interactions with persisting northern and cold-climate species will occur at the ecoregional scale [8, 77]. These areas include the mountains of southcentral Alaska and the western Brooks Range, as well as the Beaufort Coastal Plain. The strength of competition between new sets of species has not been quantified in most cases, although recent isotopic evidence suggests that realized foraging niche spaces of sympatric species are plastic enough to allow for their coexistence [83]. While this was true for 2–3 species, in regions with increasing species richness, if resources cannot be partitioned efficiently among new, increasingly saturated communities, poor competitors may suffer local extirpation [84]. Given current climate trajectories, the eventual replacement of native cold-climate and northern species by advancing interior, southern, or continental would be the likely result [84].

### Management Implications

Climate change is causing the transition of biomes, resulting in the decline of species that reside in fading habitats, especially alpine and coastal tundra [12]. Model results presented here depict the projected future distributions and composition of small mammal communities, including where biodiversity hot- and cold-spots are likely to exist in the future. Management decisions aimed at conserving biodiversity should be cognizant of where these important areas are likely to occur and which species may assemble to form the communities in these regions. To conserve biodiversity and the ecosystem services associated with the occupancy of a range of niches, it is vital to build management strategies that are based not just on current biodiversity conditions, but which incorporate projected future changes in biodiversity into such decisions [21, 85]. Potential management actions designed to conserve biodiversity and the persistence of sensitive species might include limiting road construction, extraction activities, development projects, as well as off-road recreation and disturbances in areas of high species richness.

Given that distributions and biodiversity hot-spots are projected to move northward and into mountainous areas, efforts to conserve biodiversity should consider how to facilitate the dispersal of species between current core biodiversity and projected future hotspots. Although defining and implementing biodiversity reserves is difficult and time-consuming, managing existing reserves and corridors with strategies that are cognizant of future trends in biodiversity movement is essential. Providing for core reserve areas along elevational, inland, and latitudinal gradients [21, 49, 86] may allow for the persistence of species long enough so that adaptive traits can have a chance to benefit populations [8, 60].

In Alaska where mountain ranges and the expansive Yukon River system bisect the state, geographic-scale corridors that can aid in movement across dispersal filters are already limited. Potentially important corridors identified in our models include the Nulato Hills, which provide connectivity between the rapidly changing Y-K Delta and Seward Peninsula ecosystems. The Yukon-Tanana Upland region, noted for its high species richness, provides rare high elevation refugia between the Alaska and Brooks Ranges and is also a gateway for new species from the continental interior. The DeLong Mountains in Noatak National Preserve, and the Brooks Range foothills encompassed by the National Petroleum Reserve Alaska (NPRA) should also be recognized for their projected future biodiversity potential and these areas merit sound biodiversity-conscious conservation planning.

Ultimately, treating just the symptoms of climate change (shrinking distributions, biodiversity loss, fragmented habitat connectivity, and genetic homogenization) by conserving only existing hot-spots and corridors will do little to curb the damage to ecological systems resulting from species losses [85, 87]. Conservation science must continue to promote the minimization of climate impacts and habitat disturbance to address the root causes of declining species persistence [21]. Reducing global greenhouse gas emissions and enhancing resource conservation must be prioritized to hold back the loss of species in the Arctic and around the world.

## Supporting Information

S1 Dataset Species occurrence training dataset. Archived occurrence records of 17 species of small mammal in Alaska used to generate presence-only model training dataset. Institution catalog numbers indicate institution of origin and a unique numeric identifier. Latitude and longitude coordinates are accurate to 5 decimal places. These records were subsequently focused to exclude multiple records of a species occurring at a single location.
(XLSX)

S1 Fig Future species distribution maps. Predictive maps of the distributions of 17 species of small mammal in Alaska for 2100: a) northern red-backed vole (*Clethrionomys rutilus*), b) northern collared lemming (*Dicrostonyx groenlandicus*), c) brown lemming (*Lemmus trimucronatus*), d) long-tailed vole (*Microtus longicaudus*), e) singing vole (*Microtus miurus*), f) root vole (*Microtus oeconomus*), g) meadow vole (*Microtus pennsylvanicus*), h) yellow-cheeked vole (*Microtus pennsylvanicus*), i) cinereus shrew (*Sorex cinereus*), j) pygmy shrew (*Sorex hoyi*), k) montane shrew (*Sorex monticolus*), l) American water shrew (*Sorex palustris*), m) tundra shrew (*Sorex tundrensis*), n) barren-ground shrew (*Sorex ugyunak*), o) Alaska tiny shrew (*Sorex yukonicus*), p) northern bog-lemming (*Synaptomys borealis*), q) meadow jumping mouse (*Zapus hudsonius*). Models are based on training data points (black) collected from archived museum records of occurrence. Warm colors indicate high RIO (relative index of occurrence) values and cool colors indicate areas of lower RIO values.
(TIF)

S1 File Complementary research describing current small mammal distributions. Baltensperger AP and Huettmann F (2015) Predictive spatial niche and biodiversity hotspot models for small mammal communities in Alaska: Applying machine-learning to conservation planning. Landscape Ecol 30: 681–697.
(PDF)
