# Correction: Predicted Shifts in Small Mammal Distributions and Biodiversity in the Altered Future Environment of Alaska: An Open Access Data and Machine Learning Perspective

**DOI:** 10.1371/journal.pone.0194377

**Published:** 2018-03-12

**Authors:** A. P. Baltensperger, F. Huettmann

In Table 3, the values in the fifth column are incorrect. Please see the corrected Table 3 here.

**Table 3:** Species model change metrics. Total predicted areas of presence for each of 17 species of Alaskan small mammals in 2010 and 2100. Net change is the 2010 area subtracted from that of 2100. % change is the number of pixels changed in the presence class (net change) divided by the area of the presence class for 2010. Changes in latitude, distance to coast, and elevation were calculated by subtracting the median value in 2100 from that of 2010. Negative values for latitude, coast distance, and elevation indicate southerly, coastward, and downslope shifts, respectively.

	Presence Area 2010	Presence Area 2100	Net Δ (km^2^)	% Δ	Latitude Δ (km)	Coast Distance Δ (km)	Elevation Δ (m)	Community
*Dicrostonyx groenlandicus*	603,960	550,725	-53,235	-9	-25	8	67	cold-climate
*Microtus miurus*	589,108	377,223	-211,885	-36	130	-3	167	cold-climate
*Microtus oeconomus*	1,083,164	823,741	-259,423	-24	105	45	89	cold-climate
*Sorex ugyunak*	412,527	198,763	-213,764	-52	85	-6	-66	cold-climate
**Mean**	**672,190**	**487,613**	**-184,577**	**-30**	**74**	**11**	**64**	**cold-climate**
*Microtus longicaudus*	206,803	336,130	129,327	63	35	7	33	continental
*Sorex monticolus*	335,761	382,230	46,469	14	-595	-148	108	continental
**Mean**	**271,282**	**359,180**	**87,898**	**38**	**-280**	**-71**	**71**	**continental**
*Microtus rutilus*	803,289	609,189	-194,100	-24	135	9	105	interior
*Microtus xanthognathus*	355,644	219,628	-136,016	-38	45	32	76	interior
*Sorex cinereus*	1,192,694	1,105,717	-86,977	-7	50	12	4	interior
*Sorex hoyi*	607,161	637,943	30,782	5	130	6	21	interior
**Mean**	**739,697**	**643,119**	**-96,578**	**-16**	**90**	**15**	**51**	**interior**
*Lemmus trimucronatus*	702,596	395,636	-306,960	-44	210	20	41	northern
*Sorex tundrensis*	867,006	455,138	-411,868	-48	280	2	6	northern
*Sorex yukonicus*	418,908	259,669	-159,239	-38	75	-12	-47	northern
**Mean**	**662,837**	**370,148**	**-292,689**	**-43**	**188**	**3**	**0**	**northern**
*Microtus pennsylvanicus*	335,399	294,218	-41,181	-12	-90	-71	-1	southern
*Sorex palustris*	237,571	1,049,427	811,856	342	85	-1	-202	southern
*Sorex borealis*	532,151	979,025	446,874	84	-50	-63	-20	southern
*Zapus hudsonius*	438,181	1,010,635	572,454	131	155	12	115	southern
**Mean**	**385,826**	**833,326**	**447,501**	**136**	25	-31	-27	**southern**

This error has also caused the following errors in the text:

There are errors in the first paragraph under the subheading “Model Change between 2010 and 2100” in the Results section. The correct paragraph is: Comparisons between current and future species distribution models showed an average loss in area of 29% for all species in the cold-climate, northern, and interior community groups (Table 3). Among these groups, only the pygmy shrew (*S*. *hoyi*) experienced increases in total area (5%). In contrast, distributions of all species in the continental and southern communities increased by an average of 103%, with the only exception being that the area occupied by meadow voles (*Microtus pennsylvanicus*) decreased by 12%.

There is an error in the first sentence of the fifth paragraph under the subheading “Model Change between 2010 and 2100” in the Results section. The correct sentence is: Distributions of species in the southern community were projected to grow by an average of 136% in area between 2010 and 2100.

There is an error in the last sentence of the last paragraph under the subheading “Distribution Shifts” in the Discussion section. The correct sentence is: We predicted losses of just 9% in total collared lemming distribution by 2100, which sharply contradicts other results, but may be explained by modest habitat gains predicted for southern mountain ranges that were not predicted by Prost et al. [4].

## References

[1] Baltensperger AP, Huettmann F (2015) Predicted Shifts in Small Mammal Distributions and Biodiversity in the Altered Future Environment of Alaska: An Open Access Data and Machine Learning Perspective. PLoS ONE 10(7): e0132054 https://doi.org/10.1371/journal.pone.0132054 26207828 10.1371/journal.pone.0132054 PMC4514745

