# Supplementary figures and images for: Predicted Shifts in Small Mammal Distributions and Biodiversity in the Altered Future Environment of Alaska: An Open Access Data and Machine Learning Perspective
Source: PLoS One. 2015 Jul 24;10(7):e0132054. doi: 10.1371/journal.pone.0132054 (PMC4514745; doi:10.1371/journal.pone.0132054)

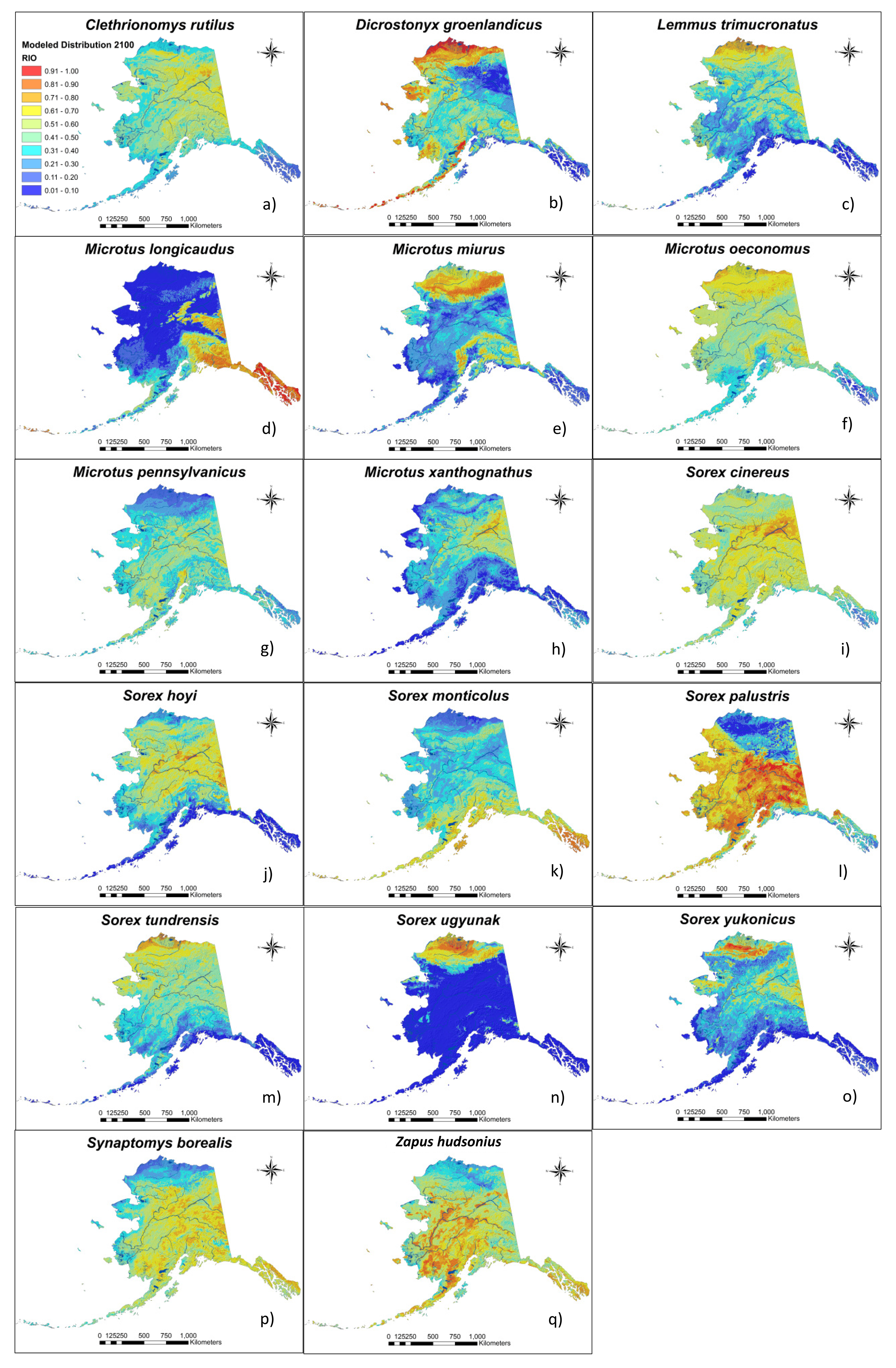

Supplement: S1 Fig — Predictive maps of the distributions of 17 species of small mammal in Alaska for 2100: a) northern red-backed vole (Clethrionomys rutilus), b) northern collared lemming (Dicrostonyx groenlandicus), c) brown lemming (Lemmus trimucronatus), d) long-tailed vole (Microtus longicaudus), e) singing vole (Microtus miurus), f) root vole (Microtus oeconomus), g) meadow vole (Microtus pennsylvanicus), h) yellow-cheeked vole (Microtus pennsylvanicus), i) cinereus shrew (Sorex cinereus), j) pygmy shrew (Sorex hoyi), k) montane shrew (Sorex monticolus), l) American water shrew (Sorex palustris), m) tundra shrew (Sorex tundrensis), n) barren-ground shrew (Sorex ugyunak), o) Alaska tiny shrew (Sorex yukonicus), p) northern bog-lemming (Synaptomys borealis), q) meadow jumping mouse (Zapus hudsonius). Models are based on training data points (black) collected from archived museum records of occurrence. Warm colors indicate high RIO (relative index of occurrence) values and cool colors indicate areas of lower RIO values. (TIF) [file pone.0132054.s002.tif]
