# Supplementary material for: Predicted Shifts in Small Mammal Distributions and Biodiversity in the Altered Future Environment of Alaska: An Open Access Data and Machine Learning Perspective
Source: PLoS One. 2015 Jul 24;10(7):e0132054. doi: 10.1371/journal.pone.0132054 (PMC4514745; doi:10.1371/journal.pone.0132054)
Supplement: S1 File — Baltensperger AP and Huettmann F (2015) Predictive spatial niche and biodiversity hotspot models for small mammal communities in Alaska: Applying machine-learning to conservation planning. Landscape Ecol 30: 681–697. (PDF) [file pone.0132054.s003.pdf]

Predictive spatial niche and biodiversity hotspot models for small mammal communities in Alaska: applying machine-learning to conservation planning

Andrew P. Baltensperger & Falk Huettmann

Landscape Ecology

ISSN 0921-2973

Landscape Ecol
DOI 10.1007/s10980-014-0150-8

ONLINE FIRST

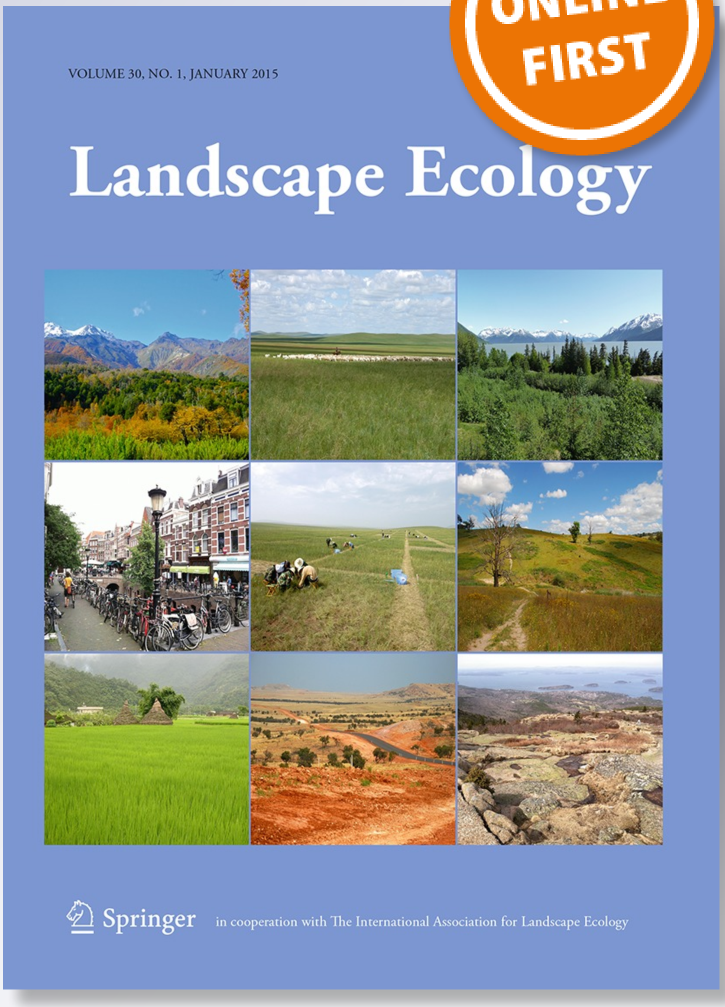

Springer

Your article is protected by copyright and all rights are held exclusively by Springer Science +Business Media Dordrecht. This e-offprint is for personal use only and shall not be self-archived in electronic repositories. If you wish to self-archive your article, please use the accepted manuscript version for posting on your own website. You may further deposit the accepted manuscript version in any repository, provided it is only made publicly available 12 months after official publication or later and provided acknowledgement is given to the original source of publication and a link is inserted to the published article on Springer's website. The link must be accompanied by the following text: "The final publication is available at link.springer.com”.

Springer

Author's personal copy

Landscape Ecol
DOI 10.1007/s10980-014-0150-8

RESEARCH ARTICLE

Predictive spatial niche and biodiversity hotspot models for small mammal communities in Alaska: applying machine-learning to conservation planning

Andrew P. Baltensperger · Falk Huettmann

Received: 6 March 2014 / Accepted: 30 December 2014
© Springer Science+Business Media Dordrecht 2015

Abstract
Context Changing global environmental conditions, especially at northern latitudes, are threatening to shift species distributions and alter wildlife communities.
Objective We aimed to establish current distributions and community arrangements of small mammals to provide important baselines for monitoring and conserving biodiversity into the future.
Methods We used 4,408 archived museum and open-access records and the machine learning algorithm, RandomForests, to create high-resolution spatial niche models for 17 species of rodents and shrews in Alaska. Models were validated using independent trapping results from 20 locations stratified along statewide mega-transects, and an average species richness curve was calculated for field samples. Community cluster analyses (*varclus*) identified geographic patterns of sympatry among species. Species models were summed to create the first small-mammal species richness map for Alaska.
Results Species richness increased logarithmically to a mean of 3.3 species per location over 1,500 trap-nights. Distribution models yielded mean accuracies of 71 % (45–90 %), and maps correctly predicted a mean of 75 % (60–95 %) of occurrences correctly in the field. Top predictors included Soil Type, Ecoregion, Landfire Land-cover, December Sea Ice, and July Temperature at the geographic scale. Cluster analysis delineated five community groups (3–4 species/group), and species richness was highest (11–13 species) over the Yukon-Tanana Uplands.
Conclusions Models presented here provide spatial predictions of current small mammal biodiversity in Alaska and an initial framework for mapping and monitoring wildlife distributions across broad landscapes into the future.

Keywords Arctic · Boreal Forest · Ecological niche modeling · Lemmings · Machine learning · Mega-transect sampling · Open-access data · RandomForests · Shrews · Voles

Electronic supplementary material The online version of this article (doi:10.1007/s10980-014-0150-8) contains supplementary material, which is available to authorized users.

A. P. Baltensperger (✉) · F. Huettmann
Department of Biology and Wildlife, University of Alaska Fairbanks, Fairbanks, AK 99775, USA
e-mail: abaltens@alaska.edu

Introduction

The arctic and boreal biomes of the circumpolar North are undergoing dramatic changes in climate, geographic distribution, ecosystem function, and food web structure (ACIA 2005; Lovejoy and Hannah 2005; IPCC 2007; Lawler et al. 2009). Mapping the current extent of spatial overlap among sympatric species will be of important conservation concern as we monitor changes in the distributions of small

Published online: 15 January 2015

Author's personal copy

mammal species in the future (Prost et al. 2013). In Alaska, small mammals are managed as non-game species under the Wildlife Action Plan (Fritts et al. 2006). This management plan recently called for the increased study of non-game and underrepresented species, especially birds and small mammals. Specific requests included efforts aimed at mapping species distributions, establishing spatial ecological system baselines, documenting biological diversity, and identifying lands vital for the conservation of wildlife in the face of increased human impacts in Alaska (Fritts et al. 2006).

In terrestrial communities, small mammals comprise a large portion of the primary consumer trophic level and represent the interface between fine-scale changes on the ground, including those related to water, soils, toxins, and micro-climate conditions (Hallett et al. 2003). Rodents are essential prey for a variety of carnivores and raptors, and also play invaluable roles in seed dispersal, nutrient cycling, plant growth, and herbivory (Newton 1979; Gough et al. 2007; Gilg et al. 2009; Olofsson et al. 2012). Insectivorous shrews, although less important as prey, are valuable in controlling invertebrate populations (Buckner 1964). Yet, despite the ecological importance of small mammals, high-resolution studies across the extent of Alaska are conspicuously lacking.

Most descriptions of small mammal distributions in Alaska have been coarse, non-quantitative, or incomplete, whereas spatially-explicit, GIS-based quantifications using modern statistical methods to analyze community composition and species richness patterns have not been conducted for the state (MacDonald and Cook 2009; Gotthardt et al. 2013; Hope et al. 2013, www.natureserve.org, www.iucnredlist.org). Using a novel niche modeling technique, we provide such a detailed, quantitative, spatial analysis that addresses many of the regional management goals for small mammals. These products should prove beneficial for land managers as they act to promote ecological stability through species diversity (Lawler et al. 2003; Hooper et al. 2005).

The ecological niche, which encompasses the environmental constraints of a species, is best suited for predicting the uncertain ecological outcome of species interactions. As conceptualized by Hutchinson (1957), the ecological niche is the space bounded by an *n*-dimensional hypervolume such that no two species can occupy exactly the same space (Cushman 2010). Dimensions include an infinite set of abiotic and biotic variables including optimal temperatures, precipitation regimes, land-cover, elevation, soil chemistry, and resource proximities, to name a few. Only by quantifying the current dimensions of niche space and interspecific overlap will it be possible to correctly predict how species may respond in a community context to a combination of altered food availability and a shifting geographic arrangement of species (Wang et al. 2004; Williams and Jackson 2007; Hope et al. 2010; Murphy et al. 2010; A. P. Baltensperger and F. Huettmann unpublished).

Spatial modeling adds the multiple dimensions of landscape space to the quantification of ecological niche breadth (Kerr et al. 2011). Beyond general linear models, machine-learning algorithms such as RandomForests, TreeNet, Mars, CART, and MaxEnt are especially adept at estimating species distributions by incorporating the environmental conditions at species' detection locations into spatial predictions (Wiersma et al. 2011). Unlike resource selection functions, which only include a limited set of variables (e.g. Johnson et al. 2004) machine-learning can include hundreds of variables and all of their interactions to identify dominant signals in the data (Breiman 2001a, b; Cutler et al. 2007). RandomForests is therefore capable of incorporating many dimensions of the ecological niche simultaneously (Cutler et al. 2007; Booms et al. 2010, Evans et al. 2011). As such, machine-learning modeling techniques are some of the newest and most comprehensive methods for deciphering complex, confounding, and non-linear relationships among variables that drive ecological processes (Breiman 2001b; Cutler et al. 2007; Kelling et al. 2009; Huettmann and Gottschalk 2010; Li et al. 2011).

To outline the potential for inter-specific competition in small mammal assemblages, we focus on the construction of detailed niche-based distribution maps for 17 species (Table 1), using them to identify the current arrangement of small mammal communities and to create a species richness map for small mammals in Alaska. This research, in concert with subsequent analyses of dietary niche overlap using stable isotopes (A. P. Baltensperger et al. unpublished) and future projections of species distributions (A. P. Baltensperger and F. Huettmann unpublished) will quantify the multi-metric ecological niche spaces occupied by small mammals, and provide projections as to how the roles of organisms are likely to shift in a future dominated by changes in climate and land-use (Wang et al. 2004).

Author's personal copy

Table 1 Integrated Taxonomic Information System (ITIS)-derived scientific names, common names, and Taxonomic Serial Numbers (TSN) for modeled species

Species	Common name	TSN #
Clethrionomys rutilus	northern red-backed vole	180293
Dicrostonyx groenlandicus	northern collared lemming	180328
Lemmus trimucronatus	brown lemming	180320
Microtus longicaudus	long-tailed vole	180299
Microtus miurus	singing vole	180309
Microtus oeconomus	root/tundra vole	180298
Microtus pennsylvanicus	meadow vole	180297
Microtus xanthognathus	yellow-cheeked/taiga vole	180301
Sorex cinereus	cinereus/masked shrew	179929
Sorex hoyi	pygmy shrew	179946
Sorex monticolus	montane/dusky shrew	179950
Sorex palustris	American water shrew	179933
Sorex tundrensis	tundra shrew	179957
Sorex ugyunak	barren-ground shrew	552509
Sorex yukonicus	Alaska tiny shrew	555663
Synaptomys borealis	northern bog-lemming	180323
Zapus hudsonius	meadow jumping mouse	180386

Methods

Study area

Alaska covers an area of 1.7 million km^2 and extends from 71.4°N latitude at Pt. Barrow to 54.2°N at Amatignak Island, and 130.0°W longitude in Portland Canal in the Alexander Archipelago to 172.4°E on Attu Island in the Aleutian Archipelago. The state contains a diversity of geographic features including several mountain ranges—notably the Alaska, Brooks, Coastal, Aleutian, and Chugach Ranges (Fig. 1a)—and elevations up to 6,036 m. Alaska's vast land area contains hundreds of glaciers and thousands of lakes that are drained by several large river systems (Molina 2001). Extreme variations in climate and geography have resulted in diverse ecosystems that include: arctic sedge tundra, boreal forest, deciduous hardwoods, peat wetlands, temperate rainforest, coastal grasslands, alpine tundra, shrub-lands, and others (Viereck et al. 1992).

Data collation

We compiled records of small mammals from digital georeferenced collections totaling over 112,000 occurrence records in Alaska. A subset of these was used as training data to create distribution models for 17 species of rodents and shrews in mainland Alaska (Table 1). Data were collated from archived occurrence datasets, primarily from the Global Biodiversity Information Facility (GBIF; www.gbif.org), but also from a variety of natural history museum collections that do not necessarily serve their data to GBIF. This compiled set of presence-only records was filtered to remove duplicates, coincident detections of species at the same location, and those records without geographic precision to at least five decimals (sub-1-m accuracy). Because of the presence-only nature of archived datasets that lack a geographically stratified design, we aimed to minimize the effects of sampling bias by using only one record per species within a 2-km radius of any given location. After manually removing these inaccurate or duplicate records, a total of 4,408 unique georeferenced small mammal records collected between 1900 and 2012 remained and comprised the final model training dataset (Appendix 1—Supplementary material 1).

Field collection

As part of a larger effort to expand wildlife occurrence databases in Alaska, and to sample small mammal tissues for stable isotope analyses, we conducted 20 small mammal inventories along two mega-transects (Assogbadjo et al. 2005) across the state between 2010

Author's personal copy

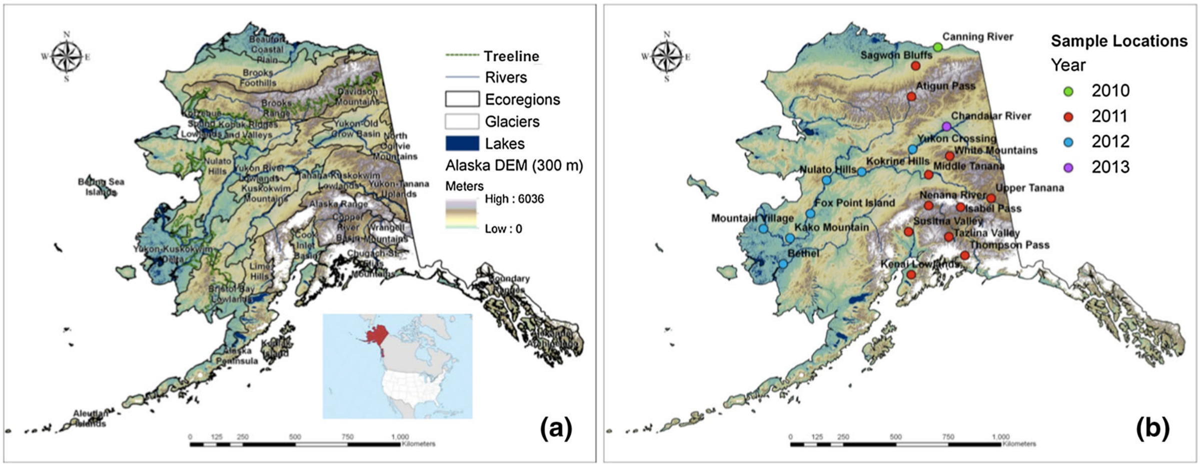

Fig. 1 Map of study areas depicting **a** Alaskan ecoregions and physical features, and **b** small mammal sampling locations between 2010 and 2013. Locations are organized along latitudinal and longitudinal megatransects across the state

and 2013 (Fig. 1b). During 2011, we sampled small mammal diversity along a 1,500-km latitudinal transect between the Arctic Ocean and the Gulf of Alaska. In 2012, we completed small mammal biodiversity sampling at seven locations along a longitudinal transect of the Yukon River during a 1,250-km canoe expedition from the Dalton Highway to Mountain Village. Additional sampling was conducted at the mouth of the Canning River on the Arctic coast during 2010 and near the mouth of the Chandalar River in 2013.

At each location we attempted to detect rodents and shrews using 200–300 traps (Sherman live traps, Museum Special snap traps, and pitfall traps) set at 10-m intervals along two or three trap-loops throughout available habitats within 1 km of the plot center. Traps remained open for five nights at each site (10 nights at Canning River) so that all sites were sampled with at least 1,500 trap-nights (number of traps × number of nights). Different trap types have different detectability rates, but the diversity of traps allowed for the sampling of a variety of taxa; Sherman live-traps primarily captured rodents, Museum Special traps captured rodents and some shrews, and pitfall traps captured only shrews. We received International Animal Care & Use (IACUC) approvals (172650-2, 172650-16) and Alaska Department of Fish and Game (ADF&G) Collection Permits (10–135, 11–114, 12–106, 13–162) for all field protocols, and specimens were archived at the University of Alaska Museum of the North.

We recorded the species detected at sampling locations for each day and plotted the accumulated species richness against the cumulative number of trap-nights. Linked with predictive modeling, this mobile, low-impact sampling scheme was designed as an efficient and cost-effective means of independently sampling biological diversity across a large geographic extent. Detections of small mammals in these surveys were later used to independently validate the accuracy of species distribution models created from the small mammal training dataset.

Model development

We used RandomForests (Salford Systems, Inc., San Diego, CA, USA; www.salford-systems.com) to create spatial distribution models for each of the 17 species of mainland small mammals in Alaska. RandomForests is a machine learning software that uses binary recursive decision trees to parse data points into terminal categories that minimize within-group variance (Cutler et al. 2007; Elith et al. 2008; Supplemental Material 2). Machine learning methods are non-parametric, and are especially adept at incorporating multi-variate interactions to analyze large, datasets without consistent sampling protocols (Prasad et al. 2006; Cutler et al. 2007; Elith et al. 2008; Evans et al. 2011). As such, they are an effective means to describe and predict the complexity of ecological systems (De'ath and Fabricius 2000; Prasad et al. 2006; Evans et al. 2011; Baltensperger et al. 2013).

Author's personal copy

Results are data-driven and not fit to a priori assumptions as would be the case using frequentist, Bayesian, or maximum entropy (MaxEnt) methods (Breiman 2001a; Cutler et al. 2007; Elith et al. 2008; Phillips et al. 2006).

Presence points as well as 'pseudo-absence' points for each species were attributed with 33 environmental predictor layers (Table 2) using the intersect (*isectpntrst*) command in the free software, Geospatial Modeling Environment 7.2 (GME; H. Beyer; www.spatialecology.com/gme). Environmental predictor variables included continuous raster (60-m accuracy) and categorical polygon layers, all of which had the potential to affect the biogeography of small mammals. These effects may occur directly at the ecosystem or landscape scales (e.g. habitat, proximity to resources, topography, etc.), or indirectly at landscape or regional scales (e.g. climate, ecoregion, etc.; Table 2).

Because this was a presence-only dataset, lacking available absences, it was necessary to generate a set of pseudo-absences to represent areas where target species weren't likely to be found. Random sets of pseudo-absences resulted in inaccurate models, so pseudo-absences were instead derived from the presence locations of all other non-target species (Elith and Leathwick 2007; VanDerWal et al. 2009).

We assumed that a presence of any of the non-target species, without the coincident occurrence of the target species within a 1-km radius, represented a pseudo-absence for the target species (Elith and Leathwick 2007). Although not ideal, given potential differences in sampling among other collection efforts, this was the best available option given the limitations of presence-only datasets and has been shown to perform as well as or better than other pseudo-absence scenarios (Breiman 2001a; Elith and Leathwick 2007; VanDerWal et al. 2009).

The combined presence/pseudo-absence datasets for each species were then modeled in RandomForests (Appendix 2—Supplementary material 2). We grew each model to 1,000 trees and used all other software default settings. RandomForests then created a coded model called a 'grove,' containing the algorithm quantifying patterns in the training dataset. Aspatial performance was assessed using a set of 'out-of-bag' training points (a subset of points automatically left out of model construction; Breiman 1996). Using this out-of-bag dataset, predictive performance of each model was calculated using the area under the curve (AUC) based on the receiver-operating characteristic (ROC), which quantified the percentages of correctly-predicted presences and absences in each model (Zweig and Campbell 1993; Fielding and Bell 1997; Huettmann and Gottschalk 2010). RandomForests was also used to rank the relative importance of environmental variables in models (Supplemental Material 2).

The grove files generated by RandomForests, containing the predictive algorithm, were then applied to a regular lattice of points (also attributed with the environmental variables) spaced at 5-km intervals across Alaska. Model outputs generated relative indices of occurrence (RIO; a ranking of pixels from 0 to 1 representing the likelihood of belonging to the 'presence' class) for each point in the regular lattice based on its underlying environmental variables. For better continuous spatial visualization, RIO values were smoothed between neighboring points across the extent of the study area using the *Inverse Distance Weighting* tool with 300-m resolution in ArcGIS 10.0 (ESRI, Inc., Redlands, CA, USA) and clipped to the state coastline, yielding a spatially continuous predictive distribution raster map of each small mammal species for Alaska. All GIS models and predictor layers were archived and are freely available on the online data repository dSpace (www.dspace.org) at the University of Alaska Fairbanks Elmer E. Rasmuson Library.

Model validation

One advantage of our predictions is that they carry known accuracy estimates since they come from a consistent, testable, and transparent prediction process. We used independent field data sampled at 20 locations across Alaska to validate the spatial predictive accuracy of all maps. Observed presences and absences of species in the field were compared with model-predicted values at field locations for each species. We used a symmetric threshold of RIO $= 0.5$ for differentiating between model-predicted presences and absences and calculated the percentage of field points correctly predicted as presences and those correctly predicted as absences by each model. Using these accuracy percentages, we calculated Cohen's kappa (a statistical measure of agreement between modeled and observed values) for each species (Cohen 1960; e.g. Baltensperger et al. 2013).

Author's personal copy

Table 2 List of predictor variables used in models, type of data (raster or polygon), and their online sources

Variable name	Data type	Source
Aspect	Raster	http://ned.usgs.gov/
Distance to coastline	Raster	http://dnr.alaska.gov/SpatialUtility/SUC?cmd=vmd&layerid=56
Distance to contaminated sites	Raster	http://dec.alaska.gov/spar/csp/db_search.htm
Distance to fire	Raster	http://forestry.alaska.gov
Distance to glaciers	Raster	http://dnr.alaska.gov/SpatialUtility/SUC?cmd=extract&layerid=27
Distance to infrastructure	Raster	http://www.snap.uaf.edu/data.php
Distance to insect damage	Raster	http://forestry.alaska.gov
Distance to lakes	Raster	http://nhd.usgs.gov/
Distance to mean December sea ice	Raster	http://nsidc.org/data/nsidc-0051.html
Distance to mean June sea ice	Raster	http://nsidc.org/data/nsidc-0051.html
Distance to Permafrost	Raster	http://agdcwww.wr.usgs.gov/agdc/agdc.html
Distance to river	Raster	http://nhd.usgs.gov/
Distance to village	Raster	http://www.adfg.alaska.gov/index.cfm?adfg=maps.data
Distance to wetlands	Raster	http://www.fws.gov/wetlands/data/
Ecoregion	Polygon	http://agdc.usgs.gov/data/usgs/erosafo/ecoreg/
Elevation	Raster	http://ned.usgs.gov/
Fire year	Raster	http://forestry.alaska.gov
Insect damage year	Raster	http://forestry.alaska.gov
Landfire land cover	Polygon	http://www.landfire.gov/vegetation.php
Mean annual precipitation	Raster	http://www.prism.oregonstate.edu/
Mean annual temperature	Raster	http://www.snap.uaf.edu/data.php
Mean December precipitation	Raster	http://www.prism.oregonstate.edu/
Mean December temperature	Raster	http://www.prism.oregonstate.edu/
Mean first day of freeze	Raster	http://www.snap.uaf.edu/data.php
Mean first day of thaw	Raster	http://www.snap.uaf.edu/data.php
Mean July precipitation	Raster	http://www.prism.oregonstate.edu/
Mean July temperature	Raster	http://www.prism.oregonstate.edu/
Mean number of grow days	Raster	http://www.snap.uaf.edu/data.php
NLCD land cover	Polygon	http://www.mrlc.gov/nlcd2006.php
Slope	Raster	http://ned.usgs.gov/
Soil type	Polygon	http://www.nrcs.usda.gov/wps/portal/nrcs/site/ak/home/
Surficial geology	Polygon	http://agdc.usgs.gov/data/usgs/geology/metadata/beikman.html
Terrain	Raster	http://ned.usgs.gov/

Raster layers have a 60-m resolution

Community composition analysis

In order to identify the degree of spatial niche overlap between species, we created a set of 50,000 random points across Alaska and attributed each point with the RIO values from the 17 species models. We used the *chart.correlation* command from the *Hmisc* package (F. Harrell; https://github.com/harrelfe/Hmisc) in R 2.12.1 (R Core Team 2013) to create a correlation matrix between species. This function yielded Pearson correlation coefficients (ρ^2) for all interspecific relationships. Species-pairings with correlation coefficients $\geq$0.5 were considered to be positively correlated and likely to co-occur in space, whereas pairings with a coefficient <-0.5 were negatively correlated and unlikely to co-occur. Coefficients between 0.5 and -0.5 were regarded as uncorrelated. Clusters of correlated species were visualized in tree

Author's personal copy

form using the *varclus* command in *Hmisc*, so that we could easily identify groups of sympatric species. Using binary reclassified distribution models we also produced maps depicting the regions of Alaska where these communities are predicted to occur.

Biodiversity hotspot analysis

A composite biodiversity map was created for small mammals in Alaska by summing individual species models of known accuracies to create an implied predictive species richness map. Continuous species models were reclassified in a binary format so that cells with RIO < 0.5 (indicating the predicted absence of a species) were assigned an absolute absence value of 0, whereas cells with RIO ≥ 0.5 were assigned an absolute presence value of 1. The reclassified binary species models were summed in ArcGIS *Raster Calculator* to yield a raster whose cells indicated the total number of species predicted to occur there. We also calculated Pearson's correlation coefficient (Zar 2010) to assess the agreement between species richness values predicted by the composite biodiversity model and the number of species observed in the field.

We highlighted regions where $\geq$11 species ($\geq$85 % of maximum predicted species richness) were predicted to occur and arbitrarily designated these as biodiversity hotspots. The resultant biodiversity map was intersected with a land ownership map of Alaska to determine which government agencies and Native corporations are responsible for managing lands on which the highest levels of small-mammal species richness occur. Ownership of biodiversity hotspots was further parsed into individual management units for each managing entity and land areas were calculated for each species total.

Results

Field sampling

Over the course of 30,700 trap-nights (Fig. 2), we captured 624 small mammals belonging to 18 species at 20 locations along two geographic mega-transects spanning Alaska (Fig. 1b). Only one species (American water shrew; *Sorex palustris*) of mainland Alaskan small mammals was not detected at any location (MacDonald and Cook 2009; Fig. 2). We documented

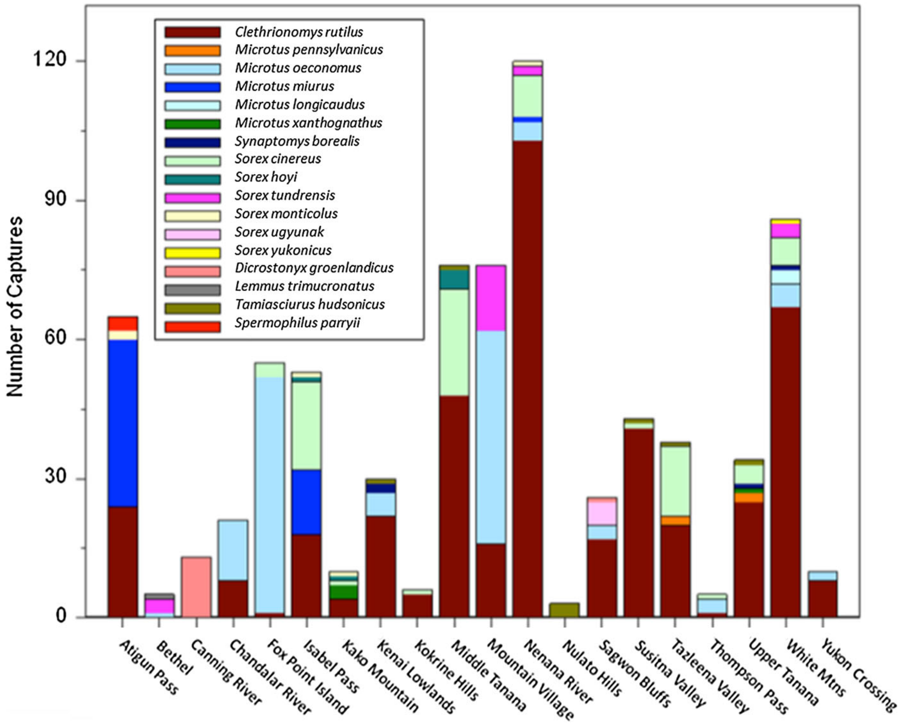

Fig. 2 Composite histogram of all species detected at 20 sampling locations between 2010 and 2013. Each location was sampled with 1,500 trap-nights

Author's personal copy

several species in regions of the state where they had not previously been identified, representing range extensions for some. These new records included the capture of the rare and understudied Alaska tiny shrew (*S. yukonicus*; but see Hope et al. 2010 for taxonomy) in the Yukon-Tanana Uplands for the first time, as well as the documentation of the westernmost occurrences of yellow-cheeked voles (*Microtus xanthognathus*) near the village of Russian Mission, and long-tailed voles (*M. longicaudus*) in the White Mountains north of Fairbanks (MacDonald and Cook 2009).

Northern red-backed voles (*Clethrionomys rutilus*; but see Carleton et al. 2014 for taxonomy) were the dominant species at all but five locations near the geographic limits of their distributions where root voles (*Microtus oeconomus*), singing voles (*M. miurus*), and northern collared lemmings (*Dicrostonyx groenlandicus*) were caught in greater abundance (Fig. 2). The dominant shrew species at most sites was the cinereus shrew (*Sorex cinereus*), except at Mountain Village where only tundra shrews (*S. tundrensis*) were captured.

Species richness curves averaged across all sites showed a roughly logarithmic increase in the number of species detected over the standard sampling period (Fig. 3). After just 300 trap-nights, a mean of 1.9 species was detected, but an additional 1,200 trap-nights resulted in the detection of fewer than two additional species and a mean total of 3.3 species per plot. However, no asymptote for species detection was attained after 1,500 trap-nights, indicating that the extent of total species richness had not been sampled.

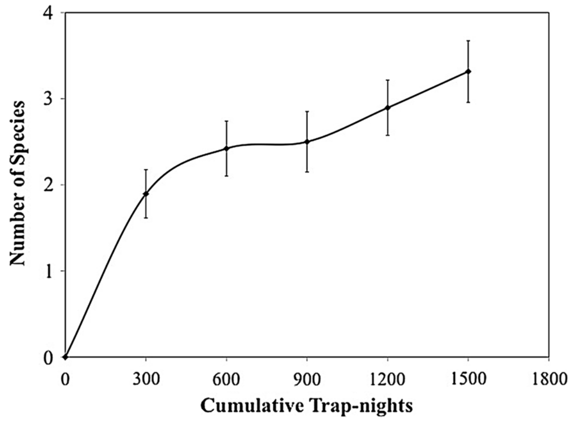

Fig. 3 Mean number of species detected at sampling locations after cumulative number of trap-nights. *Error bars* denote 95 % confidence intervals

Model accuracy

Distribution maps created from each of the 17 species models (Appendix 3—Supplementary material 3) demonstrated high degrees of accuracy when evaluated aspatially within each model using OOB cross-validation methods in RandomForests (Table 3), as well as spatially using the independent field-derived validation dataset (Table 4). Areas under the ROCs were greater than or equal to 0.90 for all species with the exceptions of water shrews and cinereus shrews (Table 3). All but two models (northern collared lemmings and northern bog-lemmings; *Synaptomys borealis*) demonstrated overall aspatial accuracies greater than 50 %. The percent of training presence points correctly identified as presences in the models (sensitivity) exceeded 90 % for 14 of the 17 species, whereas the percentages of absences correctly identified (specificity) were somewhat less accurate but nevertheless exceeded 50 % for all but two species (Table 3).

Model validation

Field validations of model predictions indicated the accurate spatial performance of most predictive models. Sensitivities and specificities were greater than or equal to 50 % for all models with the exception that just 11.1 % of cinereus shrew absences in the field were correctly identified as such by the model (Table 4). In general, sensitivities exceeded specificities, but sample sizes of presences for several species were small, making meaningful interpretation of validations difficult. A more conservative performance measure, Cohen's kappa, for long-tailed voles and singing voles was between 0.6 and 0.8, indicating 'substantial' agreement between models and field observations (Table 3; Landis and Koch 1977), whereas kappas for northern collared lemmings, root voles, montane shrews (*Sorex monticolus*), and barren-ground shrews (*S. ugyunak*) were between 0.4 and 0.6 and demonstrated 'moderate' agreement. Validations between model predictions and field detections for six other species yielded kappas between 0.2 and 0.4, and less than 0.2 for an additional four species indicating 'fair' and 'poor' agreement, respectively (Table 4). Nevertheless all models performed better than random.

Author's personal copy

Table 3 Model training dataset sample sizes and aspatial (internally cross-validated) model performance metrics for 17 species of small mammals in Alaska

Species	No. of training presences	No. of training absences	AUC ROC	Sensitivity % Presences correct	Specificity % Absences correct	Accuracy % Overall correct
Clethrionomys rutilus	949	1,157	0.96	91.15	86.34	88.51
Dicrostonyx groenlandicus	35	2,539	0.95	100.00	45.33	46.08
Lemmus trimucronatus	142	2,098	0.95	99.30	54.19	57.05
Microtus longicaudus	191	2,292	0.99	100.00	87.30	88.28
Microtus miurus	183	2,153	0.98	98.91	71.44	73.59
Microtus oeconomus	612	1,029	0.94	93.95	79.20	84.70
Microtus pennsylvanicus	244	1,725	0.96	99.18	63.30	67.75
Microtus xanthognathus	88	2,377	0.99	100.00	70.55	71.60
Sorex cinereus	818	267	0.88	70.05	85.77	73.92
Sorex hoyi	97	1,370	0.95	97.94	63.72	65.99
Sorex monticolus	566	507	0.90	80.04	83.43	81.66
Sorex palustris	13	1,701	0.77	69.23	66.20	66.22
Sorex tundrensis	195	1,071	0.94	99.49	67.13	72.12
Sorex ugyunak	37	1,634	0.99	100.00	90.33	90.54
Sorex yukonicus	34	1,610	0.98	100.00	69.88	70.50
Synaptomys borealis	142	1,986	0.91	98.59	45.92	49.44
Zapus hudsonius	72	2,348	0.95	100.00	53.58	54.96

Area under the receiver operating curve (AUC ROC) is a measure (0.00–1.00) of performance describing how well presences and absences are correctly predicted as such. Sensitivity and specificity are individual measures of correctly identified presences and absences, respectively, and accuracy is a combined measure of sensitivity and specificity

Species distributions and community compositions

Predicted distribution models of small-mammal species (Appendix 3—Supplementary material 3) were grouped by *varclus* analysis into five communities of similarly-distributed species (Fig. 4). The first community group, referred to hereafter as the 'cold-climate community', was composed of species found at high latitudes as well as high elevations mainly across the North Slope and throughout the Brooks Range (Figs. 4, 5a). None of the four species in this cluster were predicted to occur with any certainty in the center of the state throughout the central portions of the Yukon and Kuskokwim River valleys, where members of the interior and southern communities were concentrated. The second cluster, or 'northern community,' was composed of species that occurred across much of the region north of the Alaska Range (Figs. 4, 5b). These species were distributed patchily in a metapopulation arrangement across a variety of regions. Members of the third group, or 'continental community', included species occurring primarily near the Canadian border and apparently near the latitudinal extents of more southerly ranges (Figs. 4, 5c). The fourth, or 'interior community,' included two species that were both primarily restricted to a narrow swath of dry boreal forest between the Brooks and Alaska Ranges (Figs. 4, 5d). Northern red-backed voles were predicted to belong to this community, even though their range was much more expansive. The fifth species cluster, or 'southern community,' was composed of species predicted to occur mainly south of the Brooks Range (Figs. 4, 5e). Top variables were largely consistent among models and on average were ranked in the order of *Soil Type*, *Ecoregion*, *Landfire Landcover*, *December Sea Ice*, and *June Sea Ice* (Appendix 4—Supplementary material 4).

Regional biodiversity hotspots

A composite biodiversity map derived from the summation of 17 binary species models identified four main small-mammal species richness hotspots in Alaska (Fig. 5f). Model predictive accuracy, assessed

Author's personal copy

Table 4 Sample sizes and validation statistics for the independent field validation dataset compared to model predictions for 17 species of small mammals in Alaska

Species	No. of validation presences	No. of validation absences	Sensitivity % Presences correct	Specificity Absences correct	Accuracy % Overall correct	Cohen's kappa
Clethrionomys rutilus	18	2	61.11	100.00	65.00	0.24
Dicrostonyx groenlandicus	2	18	100.00	83.33	85.00	0.50
Lemmus trimucronatus	1	19	100.00	57.89	60.00	0.12
Microtus longicaudus	1	19	100.00	94.74	95.00	0.64
Microtus miurus	3	17	100.00	88.24	90.00	0.69
Microtus oeconomus	10	10	70.00	80.00	75.00	0.50
Microtus pennsylvanicus	2	18	50.00	72.22	70.00	0.12
Microtus xanthognathus	2	18	50.00	61.11	60.00	0.05
Sorex cinereus	11	9	100.00	11.11	60.00	0.12
Sorex hoyi	3	17	66.67	76.47	75.00	0.31
Sorex monticolus	4	16	75.00	81.25	80.00	0.47
Sorex palustris	0	20	–	75.00	–	–
Sorex tundrensis	4	16	75.00	56.25	60.00	0.20
Sorex ugyunak	1	19	100.00	89.47	90.00	0.46
Sorex yukonicus	1	19	100.00	73.68	75.00	0.22
Synaptomys borealis	3	17	66.67	76.47	75.00	0.31
Zapus hudsonius	1	19	100.00	78.95	80.00	0.27

Sensitivity and specificity are individual measures of correctly identified presences and absences, respectively, and accuracy is a combined measure of sensitivity and specificity. Cohen's kappa is an alternate metric that evaluates the success of observed versus modeled value agreement between −1.0 (perfect disagreement) to 1.0 (perfect agreement; Cohen 1960). Only specificity could be calculated for *Sorex palustris* because it was not detected in the field

using Pearson's correlation coefficient, indicated moderate positive correlation ($r = 0.6$) between modeled and observed species richness values for Alaska. Statewide, the majority of lands coinciding with biodiversity hotspots (>10 species) are managed by the State of Alaska (20,199 ha), and the Bureau of Land Management (BLM) and Regional Native Corporations maintain an additional 7,271 ha and 5,587 ha, respectively (Table 5). The largest and most diverse of these hotspots occurred across the Yukon-Tanana Uplands near the Canadian border. Most of this area is managed by the State of Alaska, including the largest area predicted to contain the highest statewide level of small mammal diversity (13 species) in Game Management Unit 25 (Table 5). We detected six species in 1,500 trap-nights nearby at the Upper Tanana site in 2011 (Fig. 2). A significant portion of the Yukon-Tanana Uplands hotpot also occurs on land managed by the BLM, including in the Steese National Conservation Area, where we detected seven species in 1,500 trap-nights at the White Mountains site in 2011 (Fig. 2). Doyon Regional Native Corporation, the National Park Service, and the U.S. Fish and Wildlife Service also maintain thousands of hectares containing high small mammal diversity in this region (Table 5).

The second small mammal hotspot occurred in the mountainous region between the headwaters of the Koyukuk, Kobuk, and Noatak Rivers in the central Brooks Range. Most of this land is managed by the National Park Service and the State of Alaska (Fig. 5f). A third hotspot cluster was located east of Kotzebue Sound in the Selawik National Wildlife Refuge, and on BLM and State of Alaska lands. Other diversity hotspots included several areas to the northwest of the Alaska Range in Denali National Park and on nearby BLM and State of Alaska lands (Fig. 5f). Regions predicted to contain low small mammal diversity included the North Slope, lower Yukon River, Yukon-Kuskokwim Delta, and Bristol Bay. Independent field results largely support these predictions. For example, we detected only northern collared

Author's personal copy

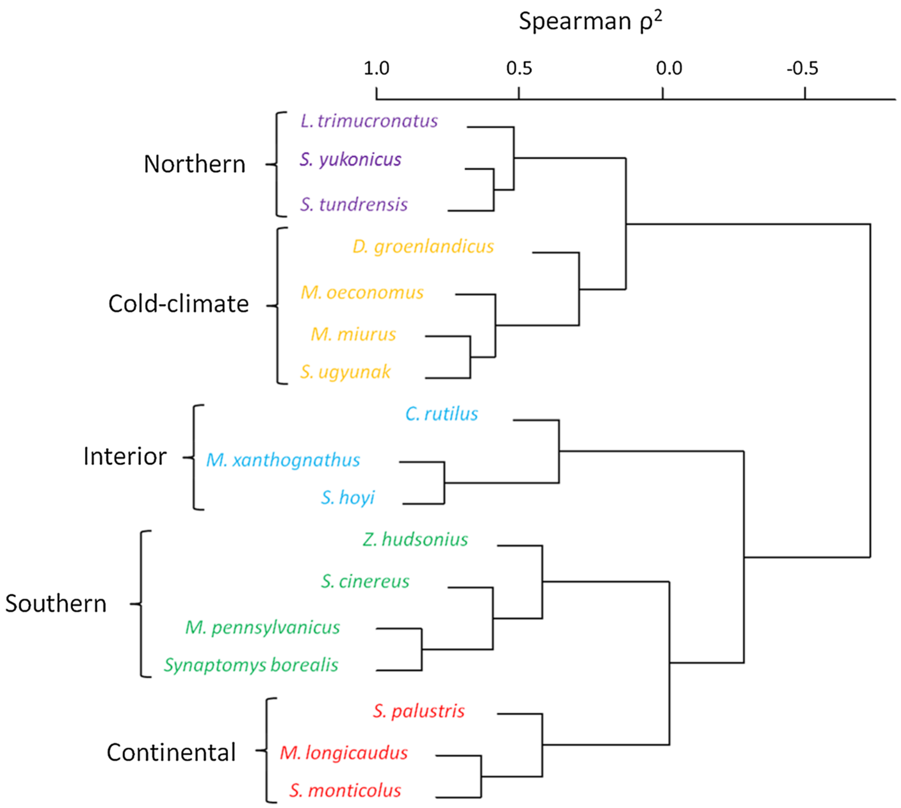

Fig. 4 Results of *varclus* analysis depicting small mammal community clusters. Species pairs with root node correlation coefficients >0.25 are considered to be part of the same community and have the same color. Species pairs with root node correlation coefficients <0 are negatively correlated

lemmings at the Canning River site on the North Slope, just two species in the Nulato Hills along the lower Yukon, and three species at Mountain Village on the Yukon-Kuskokwim Delta (Fig. 2).

Discussion

The goals of this research were to compile species occurrence records, predict species distribution and richness patterns, and to delineate the geographic community structure of small mammals in Alaska, while providing a modeling framework for other multi-species systems. We found that the distributions of the mainland small mammal species of Alaska can objectively be structured into five main community groups (Fig. 4), each with a unique set of geographic patterns (Fig. 5) but similar ecological predictors (Appendix 4—Supplementary material 4) that depict the influence of climate, soils, and vegetation on the arrangement of species across the state. We have created fine-resolution, statewide distribution maps for 17 mainland small mammal species in Alaska that represent the most accurate continuous depictions of occurrences to date (Appendix 3—Supplementary material 3). We also created species richness curves for sampling locations (Fig. 3), objective delineations of small mammal community structure (Figs. 4, 5) and a small-mammal species richness map that is the first of its kind for small mammals in Alaska (Fig. 5f). The moderate to high accuracy of these models attests to the efficiency of machine learning techniques when applied to archived datasets not collected using consistent methods.

Species richness sampling

The style of rapid assessment or 'bio-blitz' (Wilson 2006) sampling employed here allowed for small, mobile trapping teams to efficiently sample a geographically significant portion of Alaska in just two main field seasons. The detection of all but one of the small mammal species in the region is a testament to the efficacy of this design. Sampling efforts also added a large number of records to the statewide species occurrence dataset, expanding known species ranges and filling in training datasets gaps.

Author's personal copy

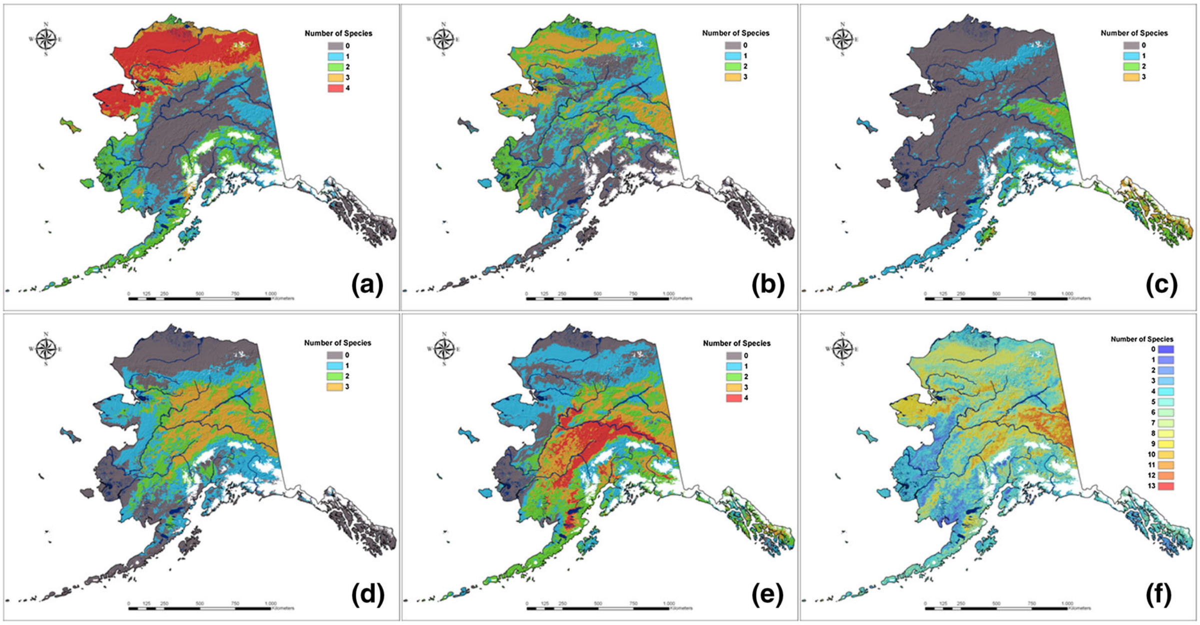

Fig. 5 Biodiversity hotspot maps depicting model-predicted small mammal species richness values for five geographic community clusters: **a** cold-climate, **b** northern, **c** continental, **d** interior, **e** southern, and **f** composite species richness map for Alaska. Maps are summations of individual species maps (Appendix 3—Supplementary material 3) converted to binary maps using relative index of occurrence (RIO) = 0.5 as a threshold to differentiate between the presence or absence of each species at each pixel

Trapping efforts detected roughly half of the model-predicted number of species occurring in most regions. The under-sampling of total species diversity at the plot level was perhaps the trade-off of a geographic mega-transect strategy designed to maximize diversity detection at the statewide scale. Despite a variety of trap styles aimed at detecting a diversity of species, and higher than average trap-nights, some species may have been especially trap-shy and remained undetected despite this intense effort. Because the number of species detected continued to increase with additional trap-nights, studies aiming to detect levels of species richness at the study site scale would be served well to trap in excess of 1,500 trap-nights.

Model progress and accuracy

All models performed remarkably well given their ability to correctly identify species presences. The models created here represent improvements in detail and accuracy over other maps for small mammals in Alaska including NatureServe and the International Union for Conservation of Nature (IUCN; www.iucnredlist.org) range maps, deductive and inductive distribution models by the Alaska Gap Analysis Project (AKGAP; http://gapanalysis.usgs.gov/species/data) models (Gotthardt et al. 2013), and other recent species niche models (Hope et al. 2013). Commonly-used range maps are coarse in scale and reflect only basic minimum convex polygon outlines of the extents of species occurrences without accounting for the influence of environmental variables in defining niche space.

The AKGAP deductive models were derived solely from habitat suitability associations and these models tended to over-predict wildlife distributions (Gotthardt et al. 2013). Although inductive AKGAP models incorporated 20 environmental variables into predictive models, this is 13 fewer than used here, and inductive models tended to under-estimate distributions. Nearly all of our species models had higher overall accuracies than models for the same species generated by AKGAP (Gotthardt et al. 2013). Our models had AUC values similar to those of AKGAP and exceeded those for the five species modeled by Hope et al. (2013).

Nevertheless, all of these ecological niche-modeling approaches provide valuable species distribution

Author's personal copy

Table 5 Land ownership status and area (ha) for biodiversity hotspots in Alaska containing at least 11 species of small mammal

Land status	Number of species			Total area (ha)
	11	12	13	
State of Alaska	11,937.4	8,225.3	36.7	20,199.4
Game Management Unit 20	9,340.9	7,471.9	4.3	16,817.1
Game Management Unit 25	887.2	705.7	32.4	1,625.3
Game Management Unit 12	666.4	87.3	0.0	753.7
Bureau of Land Management	4,953.7	2,304.9	12.1	7,270.7
Steese Conservation Area	2,515.1	1,305.8	4.7	5,089.2
Upper Black River Subunit	372.5	767.5	7.5	1,147.5
Forty-mile Planning Subunit	468.3	97.8	0.0	566.1
Native Corporations	3,611.1	1,937.5	38.8	5,587.4
Doyon Ltd	3,581.3	1,937.5	38.8	5,557.6
N.A.N.A Regional Corp	28.1	0.0	0.0	28.1
National Park Service	2,022.6	1,078.9	15.5	3,117.0
Yukon-Charley Rivers National Park	1,306.2	1,013.0	12.4	2,331.6
Denali National Park	436.2	0.4	0.0	436.6
Gates of the Arctic National Park	258.7	10.4	0.0	269.1
U.S. Fish and Wildlife Service	1,697.8	164.9	0.0	1,862.7
Arctic National Wildlife Refuge	124.0	1.9	0.0	125.9
Yukon Flats National Wildlife Refuge	109.7	155.1	0.0	264.8
Tetlin National Wildlife Refuge	159.8	0.0	0.0	159.8
Military	15.4	23.4	2.8	41.6
Private	2.6	0.0	0.0	2.6

Public lands are parsed into management subunits for geographic specificity

predictions that likely fall on a spectrum between depictions of the fundamental and realized niche spaces. Models that over-predict distributions represent more of a fundamental niche versus our models, which likely depict a more restricted realized niche. Because ecological niche models often do not include parameters to account for the details of physiology, movement, and adaptation (Bush 2002), real distributions are probably closer to some combination of these models. Future distribution mapping efforts should focus on combining several modeling approaches into a single ensemble model framework that utilizes the best components of each to produce the most accurate spatial models (Elith and Leathwick 2007; Hardy et al. 2011).

The improved accuracy of our models can be attributed to the use of more accurate presences and more representative pseudo-absence datasets based on the locations of non-target-species where target species did not also occur. This practice is an improvement over the common alternative of using randomly generated pseudo-absences or Maxent-generated absences, and resulted in more accurate models that generalized well without fitting too tightly to the training data (Elith and Leathwick 2007; Gotthardt et al. 2013). Our emphasis on correctly predicting presences may have come at the cost of reduced absence prediction, as many specificity values and consequently some overall accuracies were rather low in comparison. This effect may be a necessary detriment of using pseudo-absences in lieu of 'true absences' recorded in the field. Nevertheless, given the complexity of archived datasets, we have created models representing accurate predictions of species occurrence. We recommend using non-target surveys to aid in generating appropriate pseudo-absence scenarios for the creation of other multi-species, presence-only, distribution models.

Environmental predictors

The top three predictors, *Soil Type*, *Ecoregion*, and *Landcover*, were similar for all species, and along with some climate-related layers were consistently the most important predictors used in model algorithms (Appendix 4—Supplementary material 4). Their

Author's personal copy

prevalence demonstrates a consistent bottom-up effect of climate and soils interacting to produce habitats that drive biodiversity and community assemblage patterns. These results suggest that shifting habitat conditions resulting from changes in climate will likely have strong influence in determining distributions of wildlife and inter-specific relationships at northern latitudes.

Although these were the most important predictors on a geographic scale, their values may be overestimated at finer scales in the field. We should also note that the top three predictors are all categorical variables. Because of their categorical structure, RandomForests can easily utilize stark differences between categories to partition data points, effectively inflating the importance of these variables in the models. Nevertheless, these results provide data-mining-based foundations for more detailed hypothesis-driven analyses aimed at identifying mechanisms driving patterns of wildlife distribution.

Community structure

Small mammal species in Alaska can be organized into five main community groups that reflect their current distributions and potential for interactions with other species. *Varclus* provides a repeatable method for outlining large-scale spatial relationships among wildlife species and for documenting changes in community arrangement over time. For example, the spatial pattern for the cold-climate community is an approximate inverse prediction of the interior community's distribution, and members of these two communities do not often co-occur. As the membership of communities and the spatial arrangement between them changes with the warming climate, a consistent analysis such as *varclus* will be useful for documenting specific changes in the community composition and overlapping distributions of wildlife species around the world.

Although *varclus* community clusters indicate the most common arrangement of species at a geographic scale, they do not reflect the extent of species assemblages that may occur across different habitats. The statewide species richness map depicts overlapping distributions of $\geq$6 species over a large portion of the state, clearly demonstrating frequent cross-over between community clusters on the landscape (Fig. 5f). Correlations between species within each cluster were high, but in some cases inter-cluster correlations for some species combinations were also large. The tightest geographic relationships occurred in the southern community, and indeed all of these species were frequently detected together in the field (Fig. 2).

Trapping records included several instances in which species belonging to different community groups co-occurred at a single location. For example, northern red-backed voles, root voles, and cinereus shrews—members of three different communities—occurred together at three sampling locations (Fig. 2), indicating wider geographic niche breadths and more generalist distribution patterns. Dominant species like northern red-backed voles and cinereus shrews are increasingly being found beyond their historical distributions, leading to the higher likelihood of novel species contacts and newly emerging interspecific relationships (Hope et al. 2013). Recent stable isotope analyses have shown that in areas where species distributions overlap, dietary plasticity and niche partitioning may allow dominant and secondary species to coexist without significant competition (A.P. Baltensperger et al. unpublished). Monitoring how changes in the extent of geographic overlap between species may alter community membership can serve to identify the landscape-level effects of environmental change on wildlife persistence (Hope et al. 2013).

Regional biodiversity patterns

The region with the highest level of small mammal diversity was the Yukon-Tanana Uplands, where a maximum of 13 species were predicted to co-occur. This region appeared as a major biodiversity hotspot for several reasons. First, it is closest to the North American interior both geographically and ecologically. It is an extension of the interior Canadian boreal ecoregion and represents the farthest reach of many species that may be slowly expanding their ranges northward from the contiguous United States and Canada (Parmesan and Yohe 2003; Root et al. 2005). This includes members of the continental community, as well as members of the interior and southern communities. Many of these species are also not usually found outside of the interior and historically did not occur in Alaska prior to the last glacial maximum (MacDonald and Cook 2009; A.G. Hope personal communication).

Author's personal copy

Second, this region contains a wide range of elevations and habitats, resulting in a variety of available niches. With the diversity of habitats, it is likely that more common species such as northern red-backed voles, cinereus shrews, root voles, and other members of the northern community such as brown lemmings and tundra shrews would be found there. Because of the high elevations, the models predict that singing voles should also occur there. The only mainland species not predicted to live in this region are all three of the cold-climate species, whose distributions are far removed from this area. A similar geographic ecotone containing a variety of habitat types may also account for the hotspot in the Central Brooks Range between the headwaters of the Koyukuk, Kobuk and Noatak Rivers, as well as the hotspot cluster on the lee side of the Alaska Range. Such small mammal biodiversity hotspots occurring at the ecological crossroads along biome boundaries support the notion of these areas as important biodiversity reservoirs worthy of conservation in a changing climate (Neilson 1991).

Management implications

The conservation of biodiversity is important for a number of reasons. Although many of these species occur together across the state and appear to fill similar ecological roles, our understanding of the mechanistic functions and niche overlap of animals in ecosystems is limited (Churchfield et al. 1999; Hooper et al. 2005; Fritts et al. 2006; Prost et al. 2013). Nevertheless, apparent ecological redundancy has the benefit of insuring against the uncertainty of climate change. Maintaining a range of species that provide different ecological services and that may respond differently to environmental disturbances can have a stabilizing effect on food webs and ecosystems as they evolve (Aarssen 1997; Hooper et al. 2005; Duffy et al. 2007). Maximizing diversity also increases the likelihood that species that have disproportionately large effects on ecosystem functionality will persist (Aarssen 1997; Hooper et al. 2005). Furthermore, active conservation of a diversity of prey species occupying a variety of niches is an essential part of conserving predator diversity, and ultimately for maintaining ecosystem-wide trophic structure and functionality (Noss 1990; Lawler et al. 2009).

Because two-thirds of the land in Alaska is public, the vast majority of small-mammal hotspots occur on federal and state lands, granting an opportunity to pursue biodiversity conservation on a large scale. For land managers, the results of these types of analyses should provide them with the spatially-explicit tools and knowledge to prioritize species richness as a conservation management goal. Documenting current distribution and baseline community patterns of primary consumers at a geographic scale is the first step towards identifying the effects of impending environmental changes on the bottom-up flow of nutrients into wildlife communities (Noss 1990). Of course, species responses will not be uniform, but will depend on the capacity of each to tolerate, adapt, or disperse given rapid, large-scale ecological change (Parmesan and Yohe 2003; Williams and Jackson 2007; Hope et al. 2013). Monitoring shifts in individual species distributions over time will provide tangible accounts of how species are responding across space, and will be vital for assessing the temporal stability and adaptive capacity of natural systems (Hooper et al. 2005; Hope et al. 2013). Based on other predictive modeling efforts (Magness et al. 2008) we advocate for the establishment of a permanent network of small mammal survey sites, distributed across the state, but especially in the areas of highest diversity (e.g. Yukon Tanana Uplands), and checked at annual or decadal intervals to serve as the foundation for such long-term monitoring efforts (Noss 1990; Hope et al. 2013). Not only could a network of stations monitor species richness, but using a consistent trapping grid protocol would also allow for the calculation of species densities. These could also be modeled across space to create detailed maps of population status for multiple species. Both would be sound applications of the best professional research practices to wildlife management across a continually changing landscape.

Although species distributions and community compositions are likely to shift with the climate over time, providing wildlife with the opportunity to disperse to new areas within their niche envelope will be paramount for their persistence into the future (Bush 2002; Williams and Jackson 2007). Even as the climate, soils, and habitat conditions change, if land managers can promote the continued connectivity of important refugia along latitudinal and elevational corridors, then species incapable of coping with new environmental conditions can disperse to unexploited areas of their fundamental geographic niche (Bush 2002; Hope et al. 2013). Predicting where and how the

Author's personal copy

environment will change, determining how species are likely to respond, and conserving these areas for the future are the biggest challenges currently facing species diversity conservation worldwide.

Acknowledgments We are deeply indebted to everyone who assisted AB in the field, especially David Keiter, Edward Corp, Jessica Jordan, Alex Nicely, Casey Brown, Tim Mullet, László Kövér, and Dmitry Korobitsyn for their tireless efforts on the trap-lines. Thank you also to Link Olson, Stephen MacDonald, Joseph Cook, and GBIF for compiling and contributing valuable occurrence datasets and lending traps and equipment. Logistical support, field gear, and transportation were also generously provided by David Payer, Mark Bertram, Tom Paragi, Dashiel Feierabend, Joshua Jerome, Chris Long, Jeff Melegari, Jeremy Carlson, the Russian Mission Village Council and Kako Retreat Center. Finally, thank you to everyone who so generously offered help, advice, and fish along the Yukon River.

References

Aarssen LW (1997) High productivity in grassland ecosystems: affected by species diversity or productive species? Oikos 80:183–184

Arctic Climate Impact Assessment (2005) Impacts of a warming Arctic. Arctic Climate Impact Assessment (ACIA), Cambridge

Assogbadjo A, Sinsin B, Codjia J, Damme PV (2005) Ecological diversity and pulp, seed, and kernel production of the Baobab (*Adansonia digitata*) in Benin. Belg J Bot 138:47–56

Baltensperger AP, Mullet TC, Schmid MS, Humphries G, Kövér L, Huettmann F (2013) Seasonal observations and machine-learning-based spatial model predictions for the common raven (*Corvus corax*) in the urban, sub-arctic environment of Fairbanks, Alaska. J Polar Biol 36:1587–1599

Booms TL, Huettmann F, Schempf PF (2010) Gyrfalcon nest distribution in Alaska based on a predictive GIS model. Polar Biol 33:347–358

Breiman L (1996) Bagging predictors. Mach Learn 24:123–140

Breiman L (2001a) Random forests. Mach Learn 45:5–32

Breiman L (2001b) Statistical modeling: the two cultures. Stat Sci 16:199–231

Buckner CH (1964) Feeding behavior in four species of shrews. Can J Zool 42:259–279

Burnham K, Anderson D (2002) Model selection and multi-model inference: a practical information-theoretic approach. Springer, New York, p 488

Bush MB (2002) Distributional change and conservation on the Andean flank: a palaeoecological perspective. Clim Change Conserv Spec Issue 11:463–473

Carleton MD, Gardner AL, Pavlinov IY, Musser GG (2014) The valid generic name for red-backed voles (Muroidea: Cricetidae: Arvicolinae): restatement of the case for *Myodes* Pallas, 1811. J Mammal 95:943–959

Churchfield S, Nesterenko VA, Shvarts EA (1999) Food niche overlap and ecological separation amongst six species of coexisting forest shrews (Insectivore: Soricidae) in the Russian Far East. J Zool 248:349–359

Cohen J (1960) A coefficient of agreement for nominal scales. Educ Psychol Measur 20:37–46

Cushman SA (2010) Space and time in ecology: noise or fundamental driver? In: Cushman SA, Huettmann F (eds) Spatial complexity, informatics, and wildlife conservation. Springer, New York, p 24

Cutler DR, Edwards KH Jr, Cutler A, Hess KT, Gibson J, Lawler JJ (2007) Random forests for classification in ecology. Ecology 88:2783–2792

De'ath G, Fabricius KE (2000) Classification and regression trees: a powerful yet simple technique for ecological data analysis. Ecology 81:3178–3192

Drew CA, Wiersma YF, Huettmann F (2011) Predictive species and habitat modeling in landscape ecology. Springer, Berlin, p 314

Duffy JE, Cardinale BJ, France KE, McIntyre PB, Thébault E, Loreau M (2007) The functional role of biodiversity in ecosystems: incorporating trophic complexity. Ecol Lett 10:522–538

Elith J, Leathwick J (2007) Predicting species distributions from museum and herbarium records using multi-response models fitted with multivariate adaptive regression splines. Divers Distrib 13:265–275

Elith J, Graham CH, Anderson RP et al (2006) Novel methods improve prediction of species' distributions from occurrence data. Ecography 29:129–151

Elith J, Leathwick JR, Hastie T (2008) A working guide to boosted regression trees. J Anim Ecol 77:802–813

Evans JS, Murphy MA, Holden ZA, Cushman SA (2011) Modeling species distribution and change using Random Forest. In: Drew CA, Wiersma YF, Huettmann F (eds) Predictive species and habitat modeling in landscape ecology. Springer, Berlin, p 22

Fielding AH, Bell JF (1997) A review of methods for the assessment of prediction errors in conservation presence/absence models. Environ Conserv 234:38–49

Fritts E, Burch M, Rabe M, Schwarber J, McCracken B (2006) Our wealth maintained: a strategy for conserving Alaska's diverse wildlife and fish resources. Alaska Department of Fish and Game, Juneau, AK, p 852

Gilg O, Sittler B, Hanski I (2009) Climate change and cyclic predator–prey population dynamics in the high Arctic. Glob Change Biol 15:2634–2652

Gotthardt T, Pyare S, Huettmann F et al (2013) Predicting the range and distribution of terrestrial vertebrate species in Alaska. The Alaska Gap Analysis Project, University of Alaska, Anchorage, p 40

Gough L, Ramsey EA, Johnson DR (2007) Plant-herbivore interactions in Alaskan arctic tundra change with soil nutrient availability. Oikos 116:407–418

Hallett J, O'Connell M, Maguire C (2003) Ecological relationships of terrestrial small mammals in western coniferous forests. In: Zabel CJ, Anthony RG (eds) Mammal community dynamics: management and conservation in the coniferous forests of western North America. Cambridge University Press, New York, p 22

Hardy SM, Lindgren M, Konakanchi H, Huettmann F (2011) Predicting the distribution and ecological niche of unexploited snow crab (*Chionoecetes opilio*) populations in Alaskan waters: a first open-access ensemble model. Integr Comp Biol 51:608–622

Author's personal copy

Hastie T, Tibshirani R, Friedman JH (2001) The elements of statistical learning: data mining, inference, and prediction. Springer, New York, p 763
Hooper DU, Chapin FS, Ewel JJ et al (2005) Effects of biodiversity on ecosystem functioning: a consensus of current knowledge. Ecol Monogr 75:3–35
Hope AG, Waltari E, Dokuchaev NE et al (2010) High-latitude diversification within Eurasian least shrews and Alaska tiny shrews (Soricidae). J Mammal 91:1041–1057
Hope AG, Waltari E, Payer DC, Cook JA, Talbot SL (2013) Future distribution of tundra refugia in northern Alaska. Nat Clim Change 3:931–938
Huettmann F, Gottschalk T (2010) Simplicity, model fit, complexity and uncertainty in spatial prediction models applied over time: we are quite sure, aren't we? In: Drew CA, Wiersma YF, Huettmann F (eds) Predictive species and habitat modeling in landscape ecology. Springer, Berlin, p 31
Hutchinson GE (1957) Concluding remarks. Cold Spring Harbour Symp Quant Biol 22:415–427
IPCC (2007) Climate Change 2007: The physical science basis. In: Solomon S, Qin D, Manning M et al (eds) Contribution of Working Group I to the Fourth Assessment Report of the Intergovernmental Panel on Climate Change. Cambridge University Press, Cambridge, p 52
Johnson CJ, Seip DR, Boyce MS (2004) A quantitative approach to conservation planning: using resource selection functions to map the distribution of mountain caribou at multiple spatial scales. J Appl Ecol 41:238–251
Kelling S, Hochachka WM, Fink D et al (2009) Data-intensive science: a new paradigm for biodiversity studies. Prof Biol 59:613–620
Kerr JT, Kulkarni M, Algar A (2011) Integrating theory and predictive modeling for conservation research. In: Drew CA, Wiersma YF, Huettmann F (eds) Predictive species and habitat modeling in landscape ecology. Springer, New York, p 19
Landis JR, Koch G (1977) The measurement of observer agreement for categorical data. Biometrics 33:159–174
Lawler JJ, White D, Master LL (2003) Integrating representation and vulnerability: two approaches for prioritizing areas for conservation. Ecol Appl 13:1762–1772
Lawler JJ, Shafer SL, White D et al (2009) Projected climate-induced faunal change in the Western Hemisphere. Ecology 90:588–597
Li J, Heap AD, Potte A, Daniell JJ (2011) Application of machine learning methods to spatial interpolation of environmental variables. Environ Model Softw 26:1647–1659
Lovejoy TE, Hannah L (2005) Climate change and biodiversity. Yale University Press, New Haven, p 440
MacDonald SO, Cook JA (2009) Recent mammals of Alaska. University of Alaska Press, Fairbanks, p 387
Magness DR, Huettmann F, Morton JM (2008) Using Random Forests to provide predicted species distribution maps as a metric for ecological inventory and monitoring. In: Smolinski TG, Milanova MG, Hassanien A-E (eds) Applications of computational intelligence in biology: studies in computational intelligence. Springer, New York, p 229
Molina B (2001) Glaciers of Alaska. Alaska Geographic, 2. Alaska Geographic Society, Anchorage, p 128
Murphy K, Huettmann F, Fresco N, Morton J (2010) Connecting Alaska landscapes into the future: results from an interagency climate modeling, land management and conservation project. Fairbanks, p 100
Neilson RP (1991) Climatic contraints and issues of scale controlling regional biomes. Ecotones 1:31–51
Newton I (1979) Population ecology of raptors. A&C Black Publishers Ltd, London, p 273
Noss RF (1990) Indicators for monitoring biodiversity: a hierarchical approach. Soc Conserv Biol 4:355–364
Ohse B, Huettmann F, Ickert-Bond SM, Juday GP (2009) Modeling the distribution of white spruce (*Picea glauca*) for Alaska with high accuracy: an open access role-model for predicting tree species in last remaining wilderness areas. Polar Biol 32:1717–1729
Olofsson J, Tommervik H, Callaghan TV (2012) Vole and lemming activity observed from space. Nat Clim Change 2:880–883
Parmesan C, Yohe G (2003) A globally coherent fingerprint of climate change impacts across natural systems. Nature 42:37–42
Phillips SJ, Anderson RP, Schapire RE (2006) Maximum entropy modeling of species geographic distributions. Ecol Model 190:231–359
Prasad AM, Iverson LR, Liaw A (2006) Newer classification and regression tree techniques: bagging and random forests for ecological prediction. Ecosystems 9:181–199
Prost S, Guralnick RP, Waltari E et al (2013) Losing ground: past history and future fate of Arctic small mammals in a changing climate. Glob Change Biol 19:1–11
Root T, MacMynowski D, Mastrandrea M, Schneider S (2005) Human-modified temperatures induce species changes: joint attribution. Proc Natl Acad Sci USA 102:7465–7469
R Core Team (2013) R: a language and environment for statistical computing. R Foundation for Statistical Computing, Vienna. http://www.R-project.org/
VanDerWal J, Shoo LP, Graham C, Williams SE (2009) Selecting pseudo-absence data for presence-only distribution modeling: how far should you stray from what you know? Ecol Model 220:589–594
Viereck LA, Dyrness CT, Batten AR, Wenzlick KJ (1992) The Alaska vegetation classification. PNW-GTR-286. U.S. Department of Agriculture, Forest Service, Pacific Northwest Research Station, Portland, p 278
Wang J, Lin G, Huang J, Han X (2004) Applications of stable isotopes to study plant–animal relationships in terrestrial ecosystems. Chin Sci Bull 49:2239–2347
Wiersma YF, Huettmann F, Drew CA (2011) Landscape modeling of species and their habitats: history, uncertainty, and complexity. In: Drew CA, Wiersma YF, Huettmann F (eds) Predictive species and habitat modeling in landscape ecology. Springer, New York, p 8
Williams JW, Jackson ST (2007) Novel climates, no-analog communities, and ecological surprises. Front Ecol Environ 5:475–482
Wilson EO (2006) The creation: an appeal to save life on earth. W.W. Norton & Company Inc, New York
Zar JH (2010) Biostatistical analysis. Prentice Hall, New Jersey
Zweig MH, Campbell G (1993) Receiver-operating characteristic (ROC) plots: a fundamental evaluation tool in clinical medicine. Clin Chem 39(4):561–577
